# A Nonsynonymous/Synonymous Substitution Analysis of the B56 Gene Family Aids in Understanding B56 Isoform Diversity

**DOI:** 10.1371/journal.pone.0145529

**Published:** 2015-12-21

**Authors:** Osama Qureshi, Hyuk Cho, Madhusudan Choudhary, Joni M. Seeling

**Affiliations:** 1 Department of Biological Sciences, Sam Houston State University, Huntsville, TX, United States of America; 2 Department of Computer Science, Sam Houston State University, Huntsville, TX, United States of America; George Washington University, UNITED STATES

## Abstract

Gene duplication leads to the formation of gene families, wherein purifying or neutral selection maintains the original gene function, while diversifying selection confers new functions onto duplicated genes. The B56 gene family is highly conserved; it is encoded by one gene in protists and fungi, and five genes in vertebrates. B56 regulates protein phosphatase 2A (PP2A), an abundant heterotrimeric serine/threonine phosphatase that functions as a tumor suppressor and consists of a scaffolding “A” and catalytic “C” subunit heterodimer bound to a regulatory “B” subunit. Individual regulatory B56 subunits confer disparate functions onto PP2A in various cell-cell signaling pathways. B56 proteins share a conserved central core domain, but have divergent N- and C-termini which play a role in isoform specificity. We carried out a nonsynonymous/synonymous substitution analysis to better understand the divergence of vertebrate B56 genes. When five B56 paralogs from ten vertebrate species were analyzed, the gene family displayed purifying selection; stronger purifying selection was revealed when individual B56 isoforms were analyzed separately. The B56 core experienced stronger purifying selection than the N- and C-termini, which correlates with the presence of several contacts between the core and the AC heterodimer. Indeed, the majority of the contact points that we analyzed between B56 and the AC heterodimer experienced strong purifying selection. B56 subfamilies showed distinct patterns of selection in their N- and C-termini. The C-terminus of the B56-1 subfamily and the N-terminus of the B56-2 subfamily exhibited strong purifying selection, suggesting that these termini carry out subfamily-specific functions, while the opposite termini exhibited diversifying selection and likely carry out isoform-specific functions. We also found reduced synonymous substitutions at the N- and C-termini when grouping B56 genes by species but not by isoform, suggesting species-specific codon bias may have a role in regulating B56 gene expression.

## Introduction

The roles of kinases in signal transduction cascades are intensely studied, but much less is known about the counter-regulatory role of protein phosphatases. The human genome encodes approximately 400 serine/threonine kinases, but only about 30 serine/threonine phosphatase catalytic subunits [1]. Because of this, phosphatases were originally thought to have wide-ranging, constitutive activities. However, rather than having numerous catalytic subunits, serine/threonine phosphatases achieve diversity by forming distinct protein complexes. PP2A forms a heterotrimer with a scaffolding A subunit, a catalytic C subunit, and one of at least three different B regulatory subunit gene families (B55/PR55/B, B56/PR56/B', and B72/PR72/B'') [2]. Due to the combinatorial effects of the association of multiple subunits, and the inclusion of alternative splicing, PP2A may form as many as 200 different phosphatase complexes. B subunits are much more diverse than the A and C subunits, therefore they are the major contributors to PP2A substrate specificity and subcellular localization.

B56 proteins are highly conserved between species, sharing approximately 60% identity between human and yeast. The five human B56 paralogs (B56α, B56β, B56γ, B56δ, and B56ε) share 66% to 81% identity. B56 genes encode proteins with a highly conserved core of about 400 amino acids and variable N- and C-termini ranging from approximately ten to one hundred amino acids in humans. Even though B56 isoforms are highly conserved, they maintain distinct cellular functions, as their N- and C-termini are thought to provide isoform specificity. Alternative splicing occurs at the B56γ locus to produce transcripts with either a B56γ N-terminal extension (B56γ/γ) or a B56δ-like N-terminal extension (B56δ/γ) [3]. B56γ/γ and B56δ/γ are likely to have distinct roles in the cell, since their N-termini originate from two different B56 paralogs.

B56 carries out essential functions in numerous cell-cell signaling pathways through its role as a regulatory subunit of PP2A, and by and large these activities are isoform specific. For example, B56 isoforms modulate canonical Wnt signaling; most B56 isoforms inhibit, whereas B56ε is required for, Wnt signaling [4–6]. Casein kinase Iδ and Iε, both activators of Wnt signaling, dissociate PP2A from a protein complex that inhibits Wnt signaling [7]. Although multiple B56 subunits modulate Wnt signaling, the position at which they act varies. B56ε acts upstream of disheveled, whereas B56α acts upstream, and B56δ/γ acts downstream, of β-catenin ([4–6] and J. M. Seeling, unpublished data). B56α, but none of the other isoforms, promotes the proteasomal-mediated degradation of Myc, thereby inhibiting Myc-dependent transcription [8]. In addition to activating Wnt signaling, B56ε also positively regulates Hedgehog signaling [9]. B56β and B56δ induce neural cell differentiation through the activation of nerve growth factor (NGF) signaling [10]. Loss-of-function studies showed that B56δ, but none of the other isoforms, dephosphorylates CCAAT/enhancer binding protein β (C/EBPβ) and thereby promotes adipogenesis [11]. B56β dephosphorylates and thereby inactivates Akt, a protein kinase central to multiple growth factor signaling pathways [12]. B56γ has a direct role as a tumor suppressor, as it inhibits cell spreading and metastasis by dephosphorylating paxillin, and its loss of function cooperates with telomerase, Ras, and SV40 large T antigen to transform human cells in culture [13, 14]. In addition, mouse knockouts show that B56γ is required for heart development [15].

Protein-coding sequences can be exposed to neutral, purifying, or diversifying selection. Selective pressures may be dissimilar in distinct parts of a gene sequence, resulting in concurrent neutral, purifying, and/or diversifying selection at different positions within a gene. Gene duplication plays a vital role in organismal complexity and the evolution of new gene function [16]. Indeed, 15% of human genes likely resulted from gene duplication [17]. Diversifying selection plays a key role in the early stages after gene duplication, and twenty to thirty percent of the time, one gene of a duplicate gene pair experiences either neutral divergence or diversifying selection [18]. Subsequent to diversification, the majority of paralogs experience purifying selection to maintain their newly acquired function [19].

Most duplicate genes are lost, however, genes involved in signal transduction pathways and/or development often remain [20]. In signal transduction pathways, ligands and receptors usually evolve quickly, whereas regulatory proteins such as G proteins, kinases, and protein phosphatases generally evolve more slowly [21]. For example, the Gli gene family, which encodes a transcription factor that regulates hedgehog signaling, exhibits neutral and purifying selection in mammals [22].

We previously constructed phylogenetic trees to explore the evolution of the B56 gene family in protists, plants, fungi, and animals [23]. The B56 gene was duplicated prior to the divergence of diploblasts and triploblasts (species with two or three germ layers, respectively), and likely twice more after the divergence of echinoderms from simple chordates. These duplications and subsequent gene losses gave rise to the five B56 loci currently found in vertebrates lineages, which consist of three B56-1 subfamily genes (B56α, B56β, and B56ε) and two B56-2 subfamily genes (B56γ and B56δ). At least two vertebrate lineages have lost one of the five B56 paralogs; *Xenopus* lacks B56δ and *Aves* lacks B56β [23]. Here we explored the nonsynonymous and synonymous substitution rates within the B56 gene family of PP2A regulatory subunits to gain a deeper understanding of the five B56 vertebrate genes that differentially regulate cell-cell signaling pathways.

## Methods

### Selection and alignment of B56 gene homologs

Species with gene sequences for all five B56 paralogs (B56α, B56β, B56γ, B56δ, and B56ε), and the B56δ/γ splice variant, were selected from NCBI on the basis of high query coverage and identity toward human B56 isoforms [24]. Each isoform, with the exception of B56δ/γ, was identified in ten vertebrate species: *Danio rerio*, *Stegastes partitus*, *Alligator sinensis*, *Chrysemys picta bellii*, *Mus musculus*, *Rattus novregicus*, *Ovies aries*, *Bos taurus*, *Felis catus*, *and Homo sapiens*. B56δ/γ sequences were identified from each of the species listed above except *A*. *sinensis* and *C*. *p*. *bellii*.

Nucleotide sequences were converted into the corresponding amino acid sequences using the “nt2aa” function of MATLAB 7.11.0.584 (R2010b) (MathWorks, Natick, MA). Alignments were generated from these amino acid sequences using Clustal Omega by EMBL-EBI [25]. Clustal Omega was used as an alignment tool because of its ability to align full-length sequences with large, non-conserved terminal ends [26]. These alignments were used as a guide to insert the appropriate gaps in the original nucleotide sequences that corresponded to the aligned protein sequences. The alignment of all B56 isoforms is shown in S1 Fig.

### *dN/dS* Calculation

The nonsynonymous and synonymous substitution rates were estimated with the *dN/dS* function using the NG method of the Bioinformatics Toolbox in commercial software package MATLAB 7.11 (R2010B) (MathWorks, Natick, MA, USA). The NG method takes into account the number of synonymous and nonsynonymous substitutions and the number of potential synonymous and nonsynonymous sites [27]. *dS* substitution rates above 1.2–1.5 are considered saturated with synonymous substitutions, and may give rise to ω values which are not reliable indicators of selective pressure [28, 29]. However, overall profiles did not change markedly when ω ratios with *dS* substitution rates above 1.5 were excluded.

As the *dN/dS* function eliminated all sequences at a given position if a gap was present in one or more of the aligned sequences, less of the N- and C-termini were incorporated into alignments that included a greater number of B56 paralogs. In the analysis of all of the B56 isoforms, 20 amino acids of the N-terminus were included (19% to 63% of the N-terminus, depending on the isoform). 29 amino acids of the C-terminus were included (27% to 94% of the C-terminus). With the B56-1 subfamily, 48 amino acids of the N-terminus were included, or 72% to 91% of the N-terminus, over twice that included in the family-wide analysis. With regard to the B56-2 subfamily, 30 amino acids of the N-terminus were included, or 28% to 94% of the N-terminus, a 1.5 fold increase over the family-wide analysis. 105 amino acids of the C-terminus were included, or 99 to 100% of the C-terminus, an almost four fold increase over the family-wide analysis.

To determine the selective pressure experienced by B56 subdomains, *dN*, *dS*, and ω values were calculated using a 50 codon sliding window over the entire aligned length. *dN*, *dS*, and ω values were calculated for each gene pair, after which the values for each window were divided by the number of possible pairs to get the average value for each window. The resulting averaged *dN*, *dS*, and ω values are shown at the first codon position of each 50 codon window. Due to the window size, the last 49 amino acids of the C-termini were not plotted because the *dN*, *dS*, or ω values for the last 49 amino acids of the C-termini could not be calculated. In the single amino acid analysis, undefined *dN*/*dS* ratios in which the denominator was zero did not generate a number that could be graphed. Therefore, less than four percent of the ω ratios were graphed.

### Statistical Analysis

The Mann-Whitney Test was used in a pairwise test to determine whether the medians of the *dN*, *dS*, and ω values were significantly different [30]. Medians were used as the test statistic because the data had variable sample size, contained significant outliers, and were not normal (even after a log transformation). *p* values less than 0.05 were considered significant.

## Results and Discussion

### The vertebrate B56 gene family experienced purifying selective pressure

To better understand how five vertebrate B56 gene copies evolved into distinct genes with different functions, we analyzed the rate of nonsynonymous (*dN*) and synonymous (*dS*) substitutions during the evolution of the B56 gene family in vertebrates. We limited our analyses to ten species which possess all five B56 genes: *Danio rerio*, *Stegastes partitus*, *Alligator sinensis*, *Chrysemys picta bellii*, *Bos taurus*, *Ovis aries*, *Felis catus*, *Rattus norvegicus*, *Mus musculus*, and *Homo sapiens*. We initially examined the entire group of fifty vertebrate B56 genes. Because the N- and C-termini were not highly conserved between B56 paralogs, sequence alignments introduced more gaps in these domains than in the core domain (S1 Fig).

#### B56 family and subfamilies

**Fig 1:**
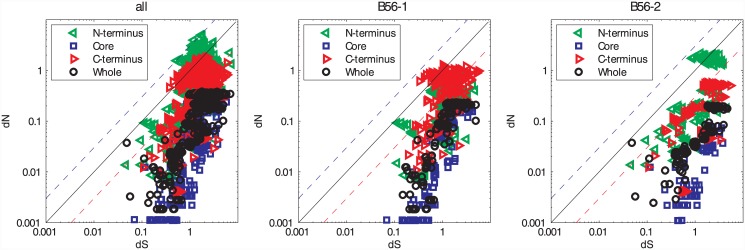
While the B56 Core Experienced Purifying Selection, the N- and C-Termini Experienced Diversifying Selection in Analyses Consisting of Multiple Paralogs. A log-log plot depicting the *dN*/*dS* values for the B56 gene family. “all” included B56α, B56β, B56γ, B56δ, and B56ε; the B56-1 subfamily included B56α, B56β, and B56ε, whereas the B56-2 subfamily included B56γ and B56δ. For each grouping, the values for the N-terminus are depicted by green triangles, the core are represented by blue squares, the C-terminus are depicted by red triangles, and the values for the entire gene are represented by black circles. The black line corresponds to *dN*/*dS* = 1 and reflects neutrality. The dashed blue line corresponds to *dN*/*dS* = 3 and the dashed red line corresponds to *dN*/*dS* = 0.3.

Each of the resultant *dN*/*dS* (ω) ratios was less than one when the entire gene was analyzed, signifying purifying selection (Fig 1 and S2 Fig). In fact, most ω ratios were less than 0.3, indicating strong purifying selection. However, one gene pair within the B56-2 subfamily, the B56γ and B56δ genes from *S*. *partitus*, exhibited close to neutral selection. This was due to the pair’s low *dS* substitution rate, as their *dN* substitution rate was similar to other gene pairs in this analysis. This low *dS* rate suggests that synonymous changes may be selected against due to codon bias in the B56-2 subfamily in *S*. *partitus*. Synonymous substitutions have been shown to alter gene expression through several mechanisms, e.g., its effects on cis regulatory elements, mRNA secondary structure, and/or rates of translation [31].

To better understand B56 subfamily evolution, we determined ω separately for the B56-1 and B56-2 subfamilies. Both subfamilies displayed strong purifying selection, as all ω ratios were less than one (Fig 1). The B56-1 and B56-2 subfamilies experienced stronger purifying selection than the entire B56 family, as would be expected if the B56-1 and B56-2 subfamilies experienced distinct forces of purifying selection (S2 Fig, S1 and S2 Tables). The synonymous substitution rate was lower in the B56-1 subfamily than the B56-2 subfamily or when all paralogs were analyzed together (S2 Fig, S1 and S2 Tables). This suggests that synonymous changes in the B56-1 subfamily may be selected against due to codon bias.

#### Individual B56 isoforms

**Fig 2:**
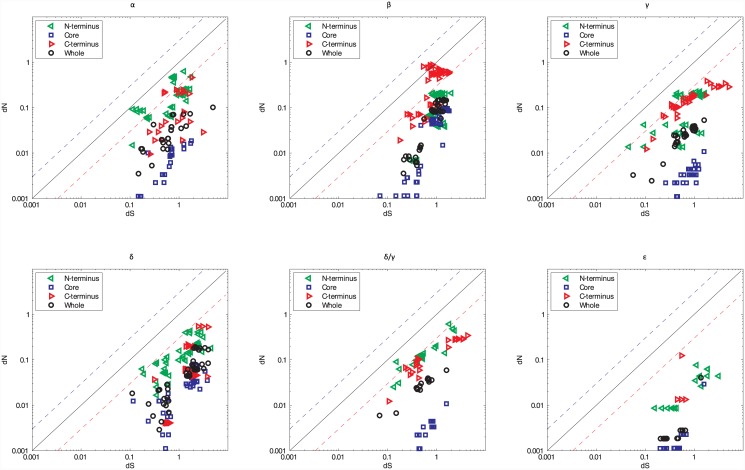
While the B56 Core Experienced Strong Purifying Selection, the N- and C-Termini Experienced Weak Purifying or Diversifying Selection When B56 Isoforms Were Analyzed Independently. A log-log plot depicting the *dN*/*dS* values for each individual B56 isoform (B56α, B56β, B56γ, B56δ/γ, B56δ, and B56ε). The values for the N-terminus are depicted by green triangular frames, the core values are represented by blue square frames, the C-terminus values are depicted by red triangular frames, and the values for the entire gene for each isoform are represented by black circular frames. The black line corresponds to *dN*/*dS* = 1 and reflects neutrality. The dashed blue line corresponds to *dN*/*dS* = 3, and the dashed red line corresponds to *dN*/*dS* = 0.3.

To specifically examine selection occurring within individual isoforms, each of the five vertebrate B56 isoforms was examined separately. These analyses included the B56δ/γ splice variant from each of the species listed previously except *A*. *sinensis* and *C*. *p*. *bellii*, where sequences of sufficient length could not be identified. Each of the isoforms displayed strong purifying selection (Fig 2). B56ε displayed an order of magnitude lower ω ratio than the other isoforms, suggesting that it experienced very strong purifying selection, or that it arose more recently (Fig 2 and S2 Fig). B56β displayed the weakest purifying selection, and the other B56 isoforms exhibited intermediate levels of purifying selection (S2 Fig). Importantly, the ω ratios from the analyses of all B56 isoforms were higher than those of individual isoforms, suggesting that the individual isoforms have undergone distinct paths of purifying selection (Figs 1 and 2, S2 Fig).

The ω plot of B56ε possessed an outlier, the *S*. *partitus* and *D*. *rerio* pairing, with an approximately five fold higher ω ratio than the other gene pairs (Fig 2). This species pair also possessed high *dN* and *dS* substitution rates for B56α, B56γ, and B56δ/γ. This may reflect reduced purifying selection of B56ε and the other isoforms in fish, due to elevated rates of substitution in fish versus mammals (and presumably other vertebrates) [32].

### The N- and C-termini experienced differential selection

It has long been hypothesized that the core, which consists primarily of α-helices and interconnecting loops, interacts with the A and C subunits of the heterotrimer, while the N- and C-termini bind the substrate and determine subcellular localization. As the core and the N- and C-termini are proposed to have distinct functions, it is likely that they may have experienced different selective pressures. We therefore analyzed *dN* and *dS* substitution rates for the core and the N- and C-terminal domains separately (S3 Table).

#### B56 family and subfamilies

When all of the B56 paralogs, the B56-1 subfamily, or the B56-2 subfamily were analyzed, the core exhibited stronger purifying selection than the whole gene, (Fig 1 and S3 Fig, S4 and S5 Tables). In addition, the core experienced stronger purifying selection in the subfamilies versus the entire gene family. The reduced ω ratios in both subfamilies as compared to the family-wide analysis suggest that significant purifying selection of the core occurred after the duplication that produced the B56-1 and B56-2 subfamily progenitors. Although the core makes contacts with the A and C subunits, portions of it are also thought to interact with substrates, and subfamily-specific substrate-binding sites may account for the reduced purifying selection in the family-wide analysis. In addition, the B56-2 core experienced stronger purifying selection than B56-1. B56-1 and B56-2 had similar dS values, so this is less likely due to the two B56-2 genes being products of a more recent duplication event than to constraints on B56-2 amino acid substitutions.

The N- and C-termini contrast with the core, showing weaker purifying selection. When all of the B56 sequences were analyzed, a significant number of gene pairs displayed ω ratios equal to or greater than one, indicating neutral or divergent selection, respectively (Fig 1). However, many ω ratios were less than one and therefore in the purifying range. The N-terminus possessed more gene pairs with strong diversifying selection than the C-terminus. The average *dN* substitution rates of the N- and C-termini were approximately five times greater than that of the core (S3–S5 Figs and S4–S9 Tables).

The pattern of selection varied between the N- and C-termini in the B56-1 and B56-2 subfamilies (Fig 1). The B56-1 subfamily displayed stronger purifying selection at its N-terminus as compared to its C-terminus, whereas the C-terminus of the B56-2 subfamily displayed stronger purifying selection than its N-terminus. The termini likely bind to molecules that differentially interact with the individual isoforms, e.g., their substrates. This suggests that subfamily-specific protein partners interact with the N-termini in the B56-1 subfamily and the C-termini in the B56-2 subfamily. On the other hand, specificity within the subfamilies was likely encoded by the C-termini in the B56-1 subfamily and the N-termini in the B56-2 subfamily, with these domains providing binding sites for paralog-specific protein partners. This finding correlates with the existence of a B56-2 mixed-isoform splice variant of the B56γ locus, B56δ/γ [3, 23]. This N-terminal alternative splicing is likely to lead to a gene product possessing a function distinct from B56γ, whereas an alternative splicing of the C-terminus would not. The presence of the B56δ/γ splice variant may have allowed the loss of the B56δ gene in *Xenopus*, as it would likely possess functions more similar to B56δ than B56γ.

#### Individual B56 isoforms

Within each B56 isoform, the strong purifying selection of the core was tempered by the weak purifying or slight diversifying selection of the N- and C-termini to yield moderately strong purifying selection of the entire gene (Fig 2). Analyses of the larger B56 groups showed diversifying selection in the termini, as the termini were likely to have been hot spots for amino acid substitutions that define the individual B56 isoforms. The N- and C-termini showed relatively similar selective pressures in each of the B56 isoforms. However, the N-termini of B56α and the C-termini of B56β had ω ratios closer to neutral, or greater than one in the case of B56β. The weak purifying or slight diversifying selection in the N-termini of B56α and the C-termini of B56β suggests that these domains were not as restricted in their amino acid substitutions as other domains of the protein or as the N- and C-termini of B56γ, B56δ, B56δ/γ, or B56ε. This also suggests that B56α and B56β possess more varied functions, or that they interact with proteins that are not as highly conserved as the proteins interacting with B56γ, B56δ, B56δ/γ, or B56ε in the species analyzed. B56β possessed the highest ω ratios for every domain except for the N-terminus, while B56ε possessed the lowest ω ratios of any other isoform (S2–S5 Figs). The strong purifying selection of B56ε may be due to the fact that it is the only isoform known to be required for Wnt signaling, while most other isoforms inhibit Wnt signaling [4–6].

The *dS* substitution rates of B56δ were approximately two-fold higher than the other paralogs. Differential *dS* substitution rates for B56δ were maintained when the whole sequence, the core, or the N-terminus were analyzed, but not the C-terminus (S2–S5 Figs; S2, S5, S7 and S9 Tables). The majority of B56 isoforms may contain regulatory elements of gene expression that fall in the coding region and are manifested in codon bias and therefore low *dS* rates. However, B56δ’s high *dS* rates suggest that its gene expression may be regulated by mechanisms that are independent of codon bias.

### Selective pressure experienced by B56 subdomains

The three dimensional structure of B56γ was determined while complexed with the PP2A AC heterodimer, as well as alone [33–35]. The core domain forms eight pseudo-HEAT repeats, each of which consists of two antiparallel α-helices of four to nineteen amino acids. The α-helical pairs are separated by five to twenty-eight amino acids, while the individual pseudo-HEAT repeats are separated by one to eleven amino acids. In these X-ray crystallography studies, the N- and C-termini were either not present in the protein that was crystallized, or were not visible in the resultant crystalline structure due to their flexible nature. The reduced purifying selection that we have shown in the termini is likely due to this flexibility. This analysis was carried out with the expectation that subdomains involved in interactions with the A and C subunits would be shared by all paralogs and therefore would have experienced strong purifying selection. However, subdomains determining paralog specificity, i.e., substrate specificity and/or subcellular localization, would have experienced weak purifying or diversifying selection. To define the selective pressures felt by discrete regions within the B56 gene family at a finer resolution, a sliding window analysis was used to examine *dN* substitution rates, *dS* substitution rates, and ω ratios.

#### B56 family and subfamilies

**Fig 3:**
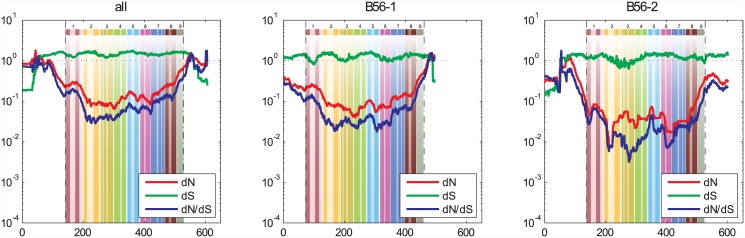
A Sliding Window Analysis for the Broader B56 Family, or the B56-1 and B56-2 Subfamilies, Revealed Selective Pressures at a Finer Level. A fifty amino acid sliding window was employed to determine *dN* (red), *dS* (green), and *dN*/*dS* (blue) values for the entire gene family (all: B56α, B56β, B56γ, B56δ, and B56ε), or the B56 subfamilies (B56-1 and B56-2). Dashed lines demarcate the core domain. The data were superimposed on a pictorial representation of the structure of the B56 conserved core domain with each number above a lightly hued bar denoting a pseudo-HEAT repeat and the darker bars within those representing α-helices.

*dS* substitution rates were between one and two throughout most of these analyses (Fig 3). When all B56 sequences were analyzed together, both the N- and C-termini provided an exception to the moderate *dS* substitution rates, as their *dS* values dropped dramatically to approximately 0.2 at the N-terminus and 0.25 at the C-terminus. In examining the B56-1 and B56-2 subfamilies, it appeared that the low *dS* substitution rate at the N-terminus of all B56 sequences was due to the low *dS* substitution rate at the N-terminus of B56-2, which had a *dS* substitution rate of 0.15. On the other hand, the low *dS* substitution rate at the C-terminus appeared to be due primarily to the 0.3 *dS* substitution rate at the C-terminus of B56-1. The low *dS* substitution rates at the termini were likely due to the effects that synonymous substitutions can have on gene expression [31]. Specifically, intragenic variation in synonymous codon usage can affect the rate of translation and therefore the dynamics of protein folding, especially for proteins containing α-helical domains [36]. The B56-2 subfamily had lower ω ratios than the B56-1 subfamily, especially in subdomains in the central core domain, signifying that specific subdomains experienced stronger purifying selection than the corresponding regions in B56-1.

#### Individual B56 isoforms

**Fig 4:**
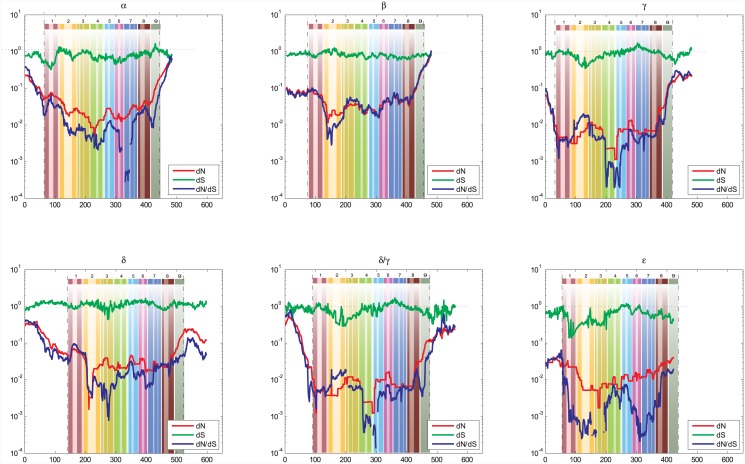
A Sliding Window Analysis of Individual B56 Isoforms Revealed Selective Pressures at a Finer Level. A fifty amino acid sliding window was employed to determine *dN* (red), *dS* (green), and *dN*/*dS* (blue) values for each individual B56 isoform (B56α, B56β, B56γ, B56δ/γ, B56δ, and B56ε). Dashed lines demarcate the core domain. The data were superimposed on a pictorial representation of the structure of the B56 conserved core domain with each number above a lightly hued bar denoting a pseudo-HEAT repeat and the darker bars within those representing α-helices.

**Fig 5:**
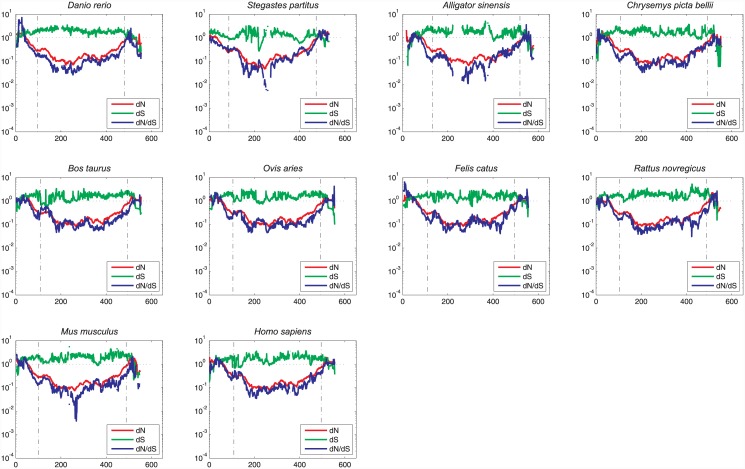
A Species-Specific Sliding Window Analysis of B56 Isoforms Revealed Species-Specific Codon Bias. A fifty amino acid sliding window was employed to determine *dN* (red), *dS* (green), and *dN*/*dS* (blue) values for five B56 paralogs (B56α, B56β, B56γ, B56δ, and B56ε) in each of ten species (*Danio rerio*, *Stegastes partitus*, *Alligator sinensis*, *Chrysemys picta bellii*, *Mus musculus*, *Rattus novregicus*, *Ovies aries*, *Bos taurus*, *Felis catus*, *and Homo sapiens*). Dashed lines demarcate the core domain. The data were superimposed on a pictorial representation of the structure of the B56 conserved core domain with each number above a lightly hued bar denoting a pseudo-HEAT repeat and the darker bars within those representing α-helices.

The dramatic drops in the *dS* substitution rates at the N- and C-termini were not observed in the B56 paralog analyses, indicating that the depressions detected in the entire B56 family may be due to species-specific codon bias (Fig 4). Indeed, *dS* substitution rates were reduced at the N- and C-termini in species-specific analyses of the B56 paralogs (Fig 5) Codon bias has been detected in vertebrates; it is often gene-specific, and tends to be observed in highly expressed genes [37, 38]. This suggests that B56 genes undergo species-specific regulation of their gene expression. The increased *dS* substitution rates of B56δ, as mentioned earlier, were also apparent. The most significant deviation of *dS* substitution rates occurred in an approximately 100 amino acid region in the N-terminal half of the B56ε core (amino acids 25–150 of the core), where it dropped down to an average of approximately 0.3. Similar depressions in *dS* substitution rates in the N-terminal end of the core were also present in B56α, B56γ, and B56δ/γ, although they were not as deep or as extensive as that in B56ε. Overall, these reductions in synonymous substitutions were most likely due to the effects that intragenic synonymous changes can have on gene expression [36].

No regions of strong purifying selection were shared by all B56 paralogs. B56γ, B56δ/γ, and B56ε displayed ω ratio depressions, indicating strong purifying selection, in pseudo-HEAT repeats one and seven; B56β, B56δ, and B56ε in repeat two; B56δ and B56ε in repeat three; B56γ and B56δ/γ in repeats four and five; all paralogs but B56β in repeat six; and B56α and B56γ in repeat eight. None of the regions of purifying selection were present in all paralogs, and the depressions did not segregate with the subfamilies. This suggests that prior to duplication, the ancestral B56 gene had multiple conserved regions that were subject either to subfamily-specific relaxation of selection or subfamily-specific selection for novel functions [16]. Alternatively, the regions of purifying selection could have arisen independently in the paralogs, or these regions could have been copied from one paralog to another through gene conversion.

### Selective pressure on individual amino acids

**Fig 6:**
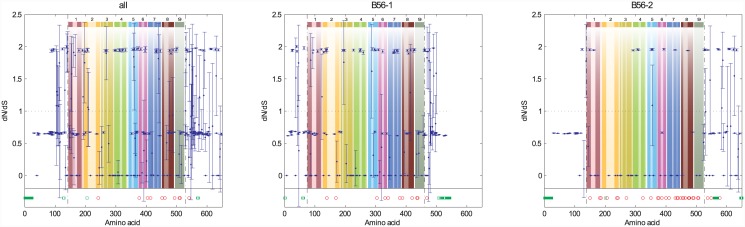
A Nonsynonymous/Synonymous Analysis of the B56 Gene Family, and B56-1 and B56-2 Subfamilies, at the Single Amino Acid Level. *dN*/*dS* values were calculated for each amino acid, and the average was plotted with 95% confidence interval brackets for each position in which ω was not undefined. Sample sets included the entire gene family (all: B56α, B56β, B56γ, B56δ, and B56ε), and the B56 subfamilies (B56-1 and B56-2). Dashed lines demarcate the core domain. The data were superimposed on a pictorial representation of the structure of the B56 conserved core domain with each number above a lightly hued bar denoting a pseudo-HEAT repeat and the darker bars within those representing α-helices. Amino acid positions in which *dN*/*dS* calculations generated an undefined number (*dS* equal to zero) that therefore could not be graphed are denoted by red circles if *dN* equaled zero, or green squares if *dN* was any number but zero.

**Fig 7:**
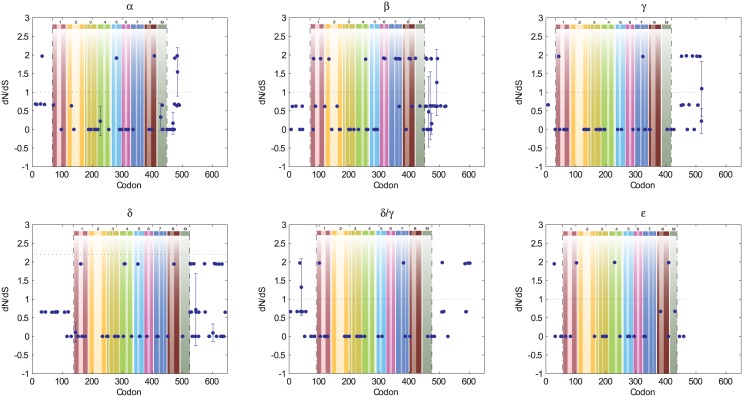
A Nonsynonymous/Synonymous Analysis of Individual B56 Paralogs at the Single Amino Acid Level. *dN*/*dS* values were calculated for each amino acid, and the average was plotted with 95% confidence interval brackets for each position in which ω was not undefined. Each B56 isoform (B56α, B56β, B56γ, B56δ/γ, B56δ, and B56ε) was analyzed. Dashed lines demarcate the core domain. The data were superimposed on a pictorial representation of the structure of the B56 conserved core domain with each number above a lightly hued bar denoting a pseudo-HEAT repeat and the darker bars within those representing α-helices. Amino acid positions in which *dN*/*dS* calculations generated an undefined number (*dS* equal to zero) that therefore could not be graphed are denoted by red circles if *dN* equaled zero, or green squares if *dN* was any number but zero.

To further delineate regions of purifying and diversifying selection in the B56 gene family, we analyzed ω ratios of individual amino acids. Whether all B56 genes, the two subfamilies, or individual isoforms were analyzed, a preponderance of three distinct ω ratios resulted: 0, 0.65, and 1.9, indicating strong purifying selection, moderate purifying selection, and diversifying selection, respectively (Figs 6 and 7). The congregation of ω ratios at 0.65 and 1.9 suggests that the residues within each of these two groups may be coevolving with one another. A study of Rubisco found that half of all residues coevolved and that the coevolving amino acids were in groups of two to sixteen residues that were localized near one another in the three dimensional structure of the protein [39]. Future studies will analyze the possibility of coevolving amino acids in B56.

As mentioned previously, B56’s pseudo-HEAT repeats consist of two antiparallel α-helices surrounding an intra-repeat loop; the pseudo-HEAT repeats are connected to one another through inter-repeat loops. Several contact points between B56γ and the PP2A AC heterodimer, primarily in the intra-repeat loops, have been identified [33, 34]. Because of the high conservation within the B56 gene family, analogous interactions are likely to occur with each B56 paralog. We found that four out of five contact points with calculable ω ratios, i.e., their *dS* substitution rates were not zero, had an ω ratio of zero (Fig 7). All but one of these was in an intra-repeat loop. These sites included PP2A A interaction sites K217 in B56α and K273 in B56ε, and PP2A C interaction sites V288 in B56β, H415 in B56δ, and K273 in B56ε. This indicated that B56 contact points with the PP2A AC heterodimer experienced intense purifying selection. However, amino acid 369 in B56β, which is in a position known to interact with PP2A C, had an ω ratio of 1.89, signifying diversifying selection. We have shown, however, that B56β experienced the weakest purifying selection of any of the paralogs (Fig 2).

## Conclusions

The B56 gene family has experienced purifying selection, however, individual paralogs experienced even stronger purifying selection, suggesting that the individual isoforms have undergone distinct paths of purifying selection. In addition, the B56 core experienced stronger purifying selection than the N- and C-termini. Within the B56-1 subfamily, the N-terminus experienced purifying selection, while the C-terminus experienced diversifying selection. This pattern was reversed in B56-2. This suggests that the N-termini carry out subfamily-specific functions and that the C-termini carry out isoform-specific functions in B56-1, with the opposite patterning in B56-2. In addition, species-specific reductions in synonymous substitutions at the N- and C-termini suggest that species-specific codon bias may play a role in regulating B56 expression. As B56 isoforms can have opposing roles in signaling pathways, understanding the basis of this antagonism may help to clarify their disparate functions. The divergence of the B56 gene family in vertebrates is likely to have provided a mechanism to finely regulate diverse cell-cell signaling pathways.

## Supporting Information

S1 Fig Sequence Alignment of all B56 Isoforms. B56α, B56β, B56γ, B56δ/γ, B56δ, and B56ε isoforms were aligned for *Danio rerio*, *Stegastes partitus*, *Alligator sinensis*, *Chrysemys picta bellii*, *Bos taurus*, *Ovis aries*, *Felis catus*, *Rattus norvegicus*, *Mus musculus*, and *Homo sapiens*.
(PDF)

S2 Fig A Nonsynonymous/Synonymous Analysis of the Whole B56 Protein. Different sets of B56 genes were grouped to analyze the selection experienced by the entire B56 gene. All B56 sequences (“all”, B56α, B56β, B56γ, B56δ, and B56ε), B56-1 and B56-2 subfamilies, as well as each B56 isoform (B56α, B56β, B56γ, B56δ/γ, B56δ, and B56ε) were analyzed. The bars represent average values, while the brackets represent a 95% confidence interval for *dN* (top), *dS* (middle), and *dN*/*dS* (bottom).
(EPS)

S3 Fig A Nonsynonymous/Synonymous Analysis of the B56 Core Domain. Different sets of B56 genes were grouped to analyze the selection experienced by the core domain of B56. All B56 sequences (“all”, B56α, B56β, B56γ, B56δ, and B56ε), B56-1 and B56-2 subfamilies, as well as each B56 isoform (B56α, B56β, B56γ, B56δ/γ, B56δ, and B56ε) were analyzed. The bars represent average values, while the brackets represent a 95% confidence interval for *dN* (top), *dS* (middle), and *dN*/*dS* (bottom).
(EPS)

S4 Fig A Nonsynonymous/Synonymous Analysis of the B56 N-Terminal Domain. Different sets of B56 genes were grouped to analyze the selection experienced by the N-terminal domain of B56. All B56 sequences (“all”, B56α, B56β, B56γ, B56δ, and B56ε), B56-1 and B56-2 subfamilies, as well as each B56 isoform (B56α, B56β, B56γ, B56δ/γ, B56δ, and B56ε) were analyzed. The bars represent average values, while the brackets represent a 95% confidence interval for *dN* (top), *dS* (middle), and *dN*/*dS* (bottom).
(EPS)

S5 Fig A Nonsynonymous/Synonymous Analysis of the C-Terminal Domain. Different sets of B56 genes were grouped to analyze the selection experienced by the C-terminal domain of B56. All B56 sequences (“all”, B56α, B56β, B56γ, B56δ, and B56ε), B56-1 and B56-2 subfamilies, as well as each B56 isoform (B56α, B56β, B56γ, B56δ/γ, B56δ, and B56ε) were analyzed. The bars represent average values, while the brackets represent a 95% confidence interval for *dN* (top), *dS* (middle), and *dN*/*dS* (bottom).
(EPS)

S6 Fig Structural Overlay of *dN* Substitution Rates Calculated on Individual Amino Acids for Each B56 Isoform. *dN* substitution rates were calculated for each amino acid, and the average was plotted with 95% confidence interval brackets. Each B56 isoform (B56α, B56β, B56γ, B56δ/γ, B56δ, and B56ε) was analyzed. Dashed lines demarcate the core domain. The data were superimposed on a pictorial representation of the structure of the B56 conserved core domain with each number above a lightly hued bar denoting a pseudo-HEAT repeat and the darker bars within those representing α-helices.
(EPS)

S7 Fig Structural Overlay of *dS* Substitution Rates Calculated on Individual Amino Acids for Each B56 Isoform. *dS* substitution rates were calculated for each amino acid, and the average was plotted with 95% confidence interval brackets. Each B56 isoform (B56α, B56β, B56γ, B56δ/γ, B56δ, and B56ε) was analyzed. Dashed lines demarcate the core domain. The data were superimposed on a pictorial representation of the structure of the B56 conserved core domain with each number above a lightly hued bar denoting a pseudo-HEAT repeat and the darker bars within those representing α-helices.
(EPS)

S1 Table *dN*, *dS*, and *dN*/*dS* Values for the Analysis of the Entire B56 Gene. The means and standard deviations from *dN*/*dS* analyses for the family-wide, B56-1, B56-2, and individual isoform groupings are provided.
(DOCX)

S2 Table *p* Values for the Analysis of the Entire B56 Gene. *p* values from *dN*/*dS* analyses for the family-wide, B56-1, B56-2, and individual isoform groupings are provided. *p* values less than 0.05 are highlighted in yellow.
(DOCX)

S3 Table Domain Positions in the Aligned B56 Sequences. The span of the N-terminus, core, and C-terminus for the family-wide, B56-1, B56-2, and individual isoform groupings from alignments generated in *dN*/*dS* analyses are provided.
(DOCX)

S4 Table *dN*, *dS*, and *dN*/*dS* Values for the B56 Core. The means and standard deviations from *dN*/*dS* analyses for the family-wide, B56-1, B56-2, and individual isoform groupings are provided.
(DOCX)

S5 Table *p* Values for the Analysis of the B56 Core. *p* values from *dN*/*dS* analyses for the family-wide, B56-1, B56-2, and individual isoform groupings are provided. *p* values less than 0.05 are highlighted in yellow.
(DOCX)

S6 Table *dN*, *dS*, and *dN*/*dS* Values for the B56 N-Terminus. The means and standard deviations from *dN*/*dS* analyses for the family-wide, B56-1, B56-2, and individual isoform groupings are provided.
(DOCX)

S7 Table *p* Values for the Analysis of the B56 N-Terminus. *p* values from *dN*/*dS* analyses for the family-wide, B56-1, B56-2, and individual isoform groupings are provided. *p* values less than 0.05 are highlighted in yellow.
(DOCX)

S8 Table *dN*, *dS*, and *dN*/*dS* Values for the B56 C-Terminus. The means and standard deviations from *dN*/*dS* analyses for the family-wide, B56-1, B56-2, and individual isoform groupings are provided.
(DOCX)

S9 Table *p* Values for the Analysis of the B56 C-Terminus. *p* values from *dN*/*dS* analyses for the family-wide, B56-1, B56-2, and individual isoform groupings are provided. *p* values less than 0.05 are highlighted in yellow.
(DOCX)

## References

[1] Shi Y. Serine/threonine phosphatases: mechanism through structure. Cell. 2009;139(3):468–84. Epub 2009/11/03. S0092-8674(09)01254-9 [pii] 10.1016/j.cell.2009.10.006 . 19879837

[2] Yang J, Phiel C. Functions of B56-containing PP2As in major developmental and cancer signaling pathways. Life Sci. 2010;87(23–26):659–66. Epub 2010/10/12. S0024-3205(10)00438-8 [pii] 10.1016/j.lfs.2010.10.003 20934435 PMC2993835

[3] Baek S, Seeling JM. Identification of a novel conserved mixed-isoform B56 regulatory subunit and spatiotemporal regulation of protein phosphatase 2A during *Xenopus laevis* development. BMC Dev Biol. 2007;7:139 . 18093315 10.1186/1471-213X-7-139 PMC2257934

[4] Seeling JM, Miller JR, Gil R, Moon RT, White R, Virshup DM. Regulation of beta-catenin signaling by the B56 subunit of protein phosphatase 2A. Science. 1999;283(5410):2089–91. . 10092233 10.1126/science.283.5410.2089

[5] Li X, Yost HJ, Virshup DM, Seeling JM. Protein phosphatase 2A and its B56 regulatory subunit inhibit Wnt signaling in *Xenopus* . Embo J. 2001;20(15):4122–31. . 11483515 10.1093/emboj/20.15.4122 PMC149155

[6] Yang J, Wu J, Tan C, Klein PS. PP2A:B56epsilon is required for Wnt/beta-catenin signaling during embryonic development. Development. 2003;130(23):5569–78. . 14522869 10.1242/dev.00762

[7] Gao ZH, Seeling JM, Hill V, Yochum A, Virshup DM. Casein kinase I phosphorylates and destabilizes the beta-catenin degradation complex. Proc Natl Acad Sci U S A. 2002;99(3):1182–7. . 11818547 10.1073/pnas.032468199 PMC122164

[8] Arnold HK, Sears RC. Protein phosphatase 2A regulatory subunit B56alpha associates with c-myc and negatively regulates c-myc accumulation. Mol Cell Biol. 2006;26(7):2832–44. . 16537924 10.1128/MCB.26.7.2832-2844.2006 PMC1430332

[9] Rorick AM, Mei W, Liette NL, Phiel C, El-Hodiri HM, Yang J. PP2A:B56epsilon is required for eye induction and eye field separation. Dev Biol. 2007;302(2):477–93. Epub 2006/11/01. . 17074314 10.1016/j.ydbio.2006.10.011

[10] Van Kanegan MJ, Strack S. The protein phosphatase 2A regulatory subunits B'beta and B'delta mediate sustained TrkA neurotrophin receptor autophosphorylation and neuronal differentiation. Mol Cell Biol. 2009;29(3):662–74. Epub 2008/11/26. 10.1128/MCB.01242-08 19029245 PMC2630673

[11] Park M, Choi YA, Lee HG, Kim KI, Lim JS, Lee MS, et al Dephosphorylation of CCAAT/enhancer-binding protein beta by protein phosphatase 2A containing B56delta is required at the early time of adipogenesis. Biochim Biophys Acta. 2014;1841(11):1608–18. Epub 2014/08/26. . 25152162 10.1016/j.bbalip.2014.08.008

[12] Rodgers JT, Vogel RO, Puigserver P. Clk2 and B56beta mediate insulin-regulated assembly of the PP2A phosphatase holoenzyme complex on Akt. Mol Cell. 2011;41(4):471–9. Epub 2011/02/19. S1097-2765(11)00090-6 [pii] 10.1016/j.molcel.2011.02.007 21329884 PMC3060660

[13] Ito A, Kataoka TR, Watanabe M, Nishiyama K, Mazaki Y, Sabe H, et al A truncated isoform of the PP2A B56 subunit promotes cell motility through paxillin phosphorylation. Embo J. 2000;19(4):562–71. . 10675325 10.1093/emboj/19.4.562 PMC305594

[14] Chen W, Possemato R, Campbell KT, Plattner CA, Pallas DC, Hahn WC. Identification of specific PP2A complexes involved in human cell transformation. Cancer Cell. 2004;5(2):127–36. . 14998489 10.1016/s1535-6108(04)00026-1

[15] Varadkar P, Despres D, Kraman M, Lozier J, Phadke A, Nagaraju K, et al The protein phosphatase 2A B56gamma regulatory subunit is required for heart development. Dev Dyn. 2014;243(6):778–90. Epub 2014/01/16. 10.1002/dvdy.24111 . 24425002

[16] Ohno S. Evolution by Gene Duplication. New York: Springer-Verlag; 1970.

[17] Moore RC, Purugganan MD. The early stages of duplicate gene evolution. Proc Natl Acad Sci U S A. 2003;100(26):15682–7. Epub 2003/12/13. 10.1073/pnas.2535513100 14671323 PMC307628

[18] Conant GC, Wagner A. Asymmetric sequence divergence of duplicate genes. Genome Res. 2003;13(9):2052–8. Epub 2003/09/04. 10.1101/gr.1252603 13/9/2052 [pii]. 12952876 PMC403682

[19] Peters AE, Bavishi A, Cho H, Choudhary M. Evolutionary constraints and expression analysis of gene duplications in Rhodobacter sphaeroides 2.4.1. BMC Res Notes. 2012;5:192 Epub 2012/04/27. 10.1186/1756-0500-5-192 22533893 PMC3494609

[20] Maere S, De Bodt S, Raes J, Casneuf T, Van Montagu M, Kuiper M, et al Modeling gene and genome duplications in eukaryotes. Proc Natl Acad Sci U S A. 2005;102(15):5454–9. Epub 2005/04/01. 10.1073/pnas.0501102102 15800040 PMC556253

[21] Wang D, Liu F, Wang L, Huang S, Yu J. Nonsynonymous substitution rate (Ka) is a relatively consistent parameter for defining fast-evolving and slow-evolving protein-coding genes. Biol Direct. 2011;6:13 Epub 2011/02/24. 10.1186/1745-6150-6-13 21342519 PMC3055854

[22] Abbasi AA, Goode DK, Amir S, Grzeschik KH. Evolution and functional diversification of the GLI family of transcription factors in vertebrates. Evol Bioinform Online. 2009;5:5–13. Epub 2009/10/09. 19812723 10.4137/ebo.s2322 PMC2747127

[23] Sommer LM, Cho H, Choudhary M, Seeling JM. Evolutionary Analysis of the B56 Gene Family of PP2A Regulatory Subunits. Int J Mol Sci. 2015;16(5):10134–57. Epub 2015/05/08. 10.3390/ijms160510134 25950761 PMC4463637

[24] Altschul SF, Gish W, Miller W, Myers EW, Lipman DJ. Basic local alignment search tool. J Mol Biol. 1990;215(3):403–10. Epub 1990/10/05. 10.1016/S0022-2836(05)80360-2 . 2231712

[25] Goujon M, McWilliam H, Li W, Valentin F, Squizzato S, Paern J, et al A new bioinformatics analysis tools framework at EMBL-EBI. Nucleic Acids Res. 2010;38(Web Server issue):W695–9. Epub 2010/05/05. 10.1093/nar/gkq313 20439314 PMC2896090

[26] Pais FS, Ruy Pde C, Oliveira G, Coimbra RS. Assessing the efficiency of multiple sequence alignment programs. Algorithms Mol Biol. 2014;9(1):4 Epub 2014/03/08. 10.1186/1748-7188-9-4 24602402 PMC4015676

[27] Nei M, Gojobori T. Simple methods for estimating the numbers of synonymous and nonsynonymous nucleotide substitutions. Mol Biol Evol. 1986;3(5):418–26. Epub 1986/09/01. . 3444411 10.1093/oxfordjournals.molbev.a040410

[28] Novichkov PS, Wolf YI, Dubchak I, Koonin EV. Trends in prokaryotic evolution revealed by comparison of closely related bacterial and archaeal genomes. J Bacteriol. 2009;191(1):65–73. Epub 2008/11/04. 10.1128/JB.01237-08 18978059 PMC2612427

[29] Huerta-Cepas J, Gabaldon T. Assigning duplication events to relative temporal scales in genome-wide studies. Bioinformatics. 2011;27(1):38–45. Epub 2010/11/16. 10.1093/bioinformatics/btq609 . 21075746

[30] Mann HB, Whitney DR. On a Test of Whether one of Two Random Variables is Stochastically Larger than the Other. Annals of Mathematical Statistics. 1947;18:50–60.

[31] Hunt RC, Simhadri VL, Iandoli M, Sauna ZE, Kimchi-Sarfaty C. Exposing synonymous mutations. Trends Genet. 2014;30(7):308–21. Epub 2014/06/24. S0168-9525(14)00068-7 [pii] 10.1016/j.tig.2014.04.006 . 24954581

[32] Robinson-Rechavi M, Laudet V. Evolutionary rates of duplicate genes in fish and mammals. Mol Biol Evol. 2001;18(4):681–3. Epub 2001/03/27. . 11264421 10.1093/oxfordjournals.molbev.a003849

[33] Xu Y, Xing Y, Chen Y, Chao Y, Lin Z, Fan E, et al Structure of the protein phosphatase 2A holoenzyme. Cell. 2006;127(6):1239–51. . 17174897 10.1016/j.cell.2006.11.033

[34] Cho US, Xu W. Crystal structure of a protein phosphatase 2A heterotrimeric holoenzyme. Nature. 2007;445(7123):53–7. . 17086192 10.1038/nature05351

[35] Magnusdottir A, Stenmark P, Flodin S, Nyman T, Kotenyova T, Graslund S, et al The structure of the PP2A regulatory subunit B56 gamma: the remaining piece of the PP2A jigsaw puzzle. Proteins. 2009;74(1):212–21. Epub 2008/07/12. 10.1002/prot.22150 . 18618707

[36] Quax TE, Claassens NJ, Soll D, van der Oost J. Codon Bias as a Means to Fine-Tune Gene Expression. Mol Cell. 2015;59(2):149–61. Epub 2015/07/18. S1097-2765(15)00402-5 [pii] 10.1016/j.molcel.2015.05.035 . 26186290 PMC4794256

[37] Hershberg R, Petrov DA. Selection on codon bias. Annu Rev Genet. 2008;42:287–99. Epub 2008/11/06. 10.1146/annurev.genet.42.110807.091442 . 18983258

[38] Nabiyouni M, Prakash A, Fedorov A. Vertebrate codon bias indicates a highly GC-rich ancestral genome. Gene. 2013;519(1):113–9. Epub 2013/02/05. S0378-1119(13)00074-7 [pii] 10.1016/j.gene.2013.01.033 . 23376453

[39] Wang M, Kapralov MV, Anisimova M. Coevolution of amino acid residues in the key photosynthetic enzyme Rubisco. BMC Evol Biol. 2011;11:266 Epub 2011/09/29. 10.1186/1471-2148-11-266 21942934 PMC3190394

