# Supplementary material for: A Nonsynonymous/Synonymous Substitution Analysis of the B56 Gene Family Aids in Understanding B56 Isoform Diversity
Source: PLoS One. 2015 Dec 21;10(12):e0145529. doi: 10.1371/journal.pone.0145529 (PMC4687035; doi:10.1371/journal.pone.0145529)
Supplement: S1 Fig — B56α, B56β, B56γ, B56δ/γ, B56δ, and B56ε isoforms were aligned for Danio rerio, Stegastes partitus, Alligator sinensis, Chrysemys picta bellii, Bos taurus, Ovis aries, Felis catus, Rattus norvegicus, Mus musculus, and Homo sapiens. (PDF) [file pone.0145529.s001.pdf]

```
CLUSTAL O(1.2.1) multiple sequence alignment


gi|530584065:87-1661                ------------------------------------------------------------	0
gi|557265256:74-1648                ------------------------------------------------------------	0
gi|755745075:318-1892               ------------------------------------------------------------	0
gi|555290051:121-1695               ------------------------------------------------------------	0
gi|300798097:82-1656                ------------------------------------------------------------	0
gi|240849335:141-1715               ------------------------------------------------------------	0
gi|803180317:2708-4297              ------------------------------------------------------------	0
gi|134085945:20-1594                ------------------------------------------------------------	0
gi|688603875:111-1688               ------------------------------------------------------------	0
gi|657585651:111-1688               ------------------------------------------------------------	0
gi|657585641:75-1811                --------------------------MPNKTKKDKESVK-----------SGKVAKSGAQ	23
gi|50726891:63-1868                 --------------------------MPNKTKKEKEPTKSAKSSTKNSNA--SAGKDVGT	32
gi|426251100:1-1800                 ----------------------MAEDNAHQTPKFKEPPKLAKGTAKPSSSS----KDGGG	34
gi|14669809:89-1873                 --------------------------MSYKLKKDKEPSKLAKGTAKPSSSS----KDGGG	30
gi|672023745:791-2578               --------------------------MPYKLKKDKEPPKLAKGTAKPSSSS----KDGGG	30
gb|L76702.1|HUMB56DA:188-1996       --------------------------MPYKLKKEKEPPKVAKCTAKPSSSG----KDGGG	30
gi|329744635:107-1909               --------------------------MPYKLKKEKEPPKLAKGTAKPSSSS----KDGGG	30
gi|755728309:140-1948               --------------------------MPYKLKKEKETPKLAKGTTKPSSSG----KDGGG	30
gi|641758569:96-1892                --------------------------MPYKLKKEKEPPKSAKSTTKPSSGSSGGGKDGGA	34
gi|557295947:96-1958                MAKAGKSTTWLKWIILLNSLRGLLLHDGSINKAIQEPPKSAKSTTKSSGG--SSGKDGGA	58
gi|688564053:1400-2911              ------------------------------------------------------------	0
gi|657563479:1-1563                 ------------------------------------------------------------	0
gi|115497699:359-1849               ------------------------------------------------------------	0
gi|803191297:577-2067               ------------------------------------------------------------	0
gi|60593006:48-1541                 ------------------------------------------------------------	0
gi|142364854:598-2091               ------------------------------------------------------------	0
gb|L42374.1|HUMPP2ABA:326-1819      ------------------------------------------------------------	0
gi|587003404:628-2121               ------------------------------------------------------------	0
gi|641793336:1879-3372              ------------------------------------------------------------	0
gi|557320144:379-1860               ------------------------------------------------------------	0
gi|51491868:454-1860                ------------------------------------------------------------	0
gi|657554348:108-1517               ------------------------------------------------------------	0
gi|641761822:526-1914               ------------------------------------------------------------	0
gi|557266651:182-1570               ------------------------------------------------------------	0
gi|586990135:195-1598               ------------------------------------------------------------	0
gi|532164714:591-1994               ------------------------------------------------------------	0
gi|134085748:529-1932               ------------------------------------------------------------	0
gi|426233507:51-1454                ------------------------------------------------------------	0
gi|55741699:574-1977                ------------------------------------------------------------	0
gi|672056393:586-1989               ------------------------------------------------------------	0
gi|528511094:406-1860               ------------------------------------------------------------	0
gi|657570088:928-2469               ------------------------------------------------------------	0
```

```
gi|557292702:1-1446                ------------------------------------------------------------      0
gi|641796061:534-1967              ------------------------------------------------------------      0
gi|118130061:626-2086              ------------------------------------------------------------      0
gi|157823929:178-1638              ------------------------------------------------------------      0
gi|126165211:499-1968              ------------------------------------------------------------      0
gi|426240097:195-1664              ------------------------------------------------------------      0
gb|L42373.1|HUMPP2A:572-2032       ------------------------------------------------------------      0
gi|755806859:84-1547               ------------------------------------------------------------      0


gi|530584065:87-1661               --------------------------------------------------MLTCNKAG--      8
gi|557265256:74-1648               --------------------------------------------------MLTCNKAG--      8
gi|755745075:318-1892              --------------------------------------------------MWTCNKAG--      8
gi|555290051:121-1695              --------------------------------------------------MLTCNKAG--      8
gi|300798097:82-1656               --------------------------------------------------MLTCNKAG--      8
gi|240849335:141-1715              --------------------------------------------------MLTCNKAG--      8
gi|803180317:2708-4297             --------------------------------------------------MLTCNKAG--      8
gi|134085945:20-1594               --------------------------------------------------MLTCNKAG--      8
gi|688603875:111-1688              --------------------------------------------------MLTCS-----      5
gi|657585651:111-1688              --------------------------------------------------MLACTRPAS-      9
gi|657585641:75-1811               ENS----------------EHEQSSNRKASNSAPPT------------TQLLKGKQPGSQ      55
gi|50726891:63-1868                ENSEEAQ---------------QTASKRPSNSTPPP------------TQLNKIKYSGGP      65
gi|426251100:1-1800                ESTEEPQPPPQP--------QPPSSNKRPSNSTPPP------------TQLSKIKYSGGP      74
gi|14669809:89-1873                ENTDEAQPQP--------QSQSPSSNKRPSNSTPPP------------TQLSKIKYSGGP      70
gi|672023745:791-2578              ENTDTEA-------QPQPQPQPPSSNKRPSNSTPPP------------TQLSKIKYSGGP      71
gb|L76702.1|HUMB56DA:188-1996      ENTEEAQPQPQPQPQPQAQSQPPSSNKRPSNSTPPP------------TQLSKIKYSGGP      78
gi|329744635:107-1909              ESTEEAQPQPQPQP--QPQPQPPSSNKRPSNSTPPP------------TQLSKIKYSGGP      76
gi|755728309:140-1948              ESAEEAQPQPQPQPQPQPQPQPPSSNKRPSNSTPPP------------TQLSKIKYSGGP      78
gi|641758569:96-1892               ENSEEVQQ--------QQQPPPPTSNKRPSNSTPPP------------TQLNKIKYSGGP      74
gi|557295947:96-1958               ENSEEVQQ--------QQ--QQTTSNKRPSNSAPPP------------TQLNKIKYSGGP      96
gi|688564053:1400-2911             ---------------------METPTKLPPSSP---SSPTTGFSVPSAEKVDGFPRRSMR      36
gi|657563479:1-1563                ----------------------METTKLPPSSP---SSPTTSFSVPSAEKVDGFPRRSMR      35
gi|115497699:359-1849              -----------------------METKLPPASTPTSPSSPGLSPVPPPDKVDGFSRRSLR      37
gi|803191297:577-2067              -----------------------METKLPPASTPTSPSSPGLSPVPPPDKVDGFSRRSLR      37
gi|60593006:48-1541                -----------------------METKLPPASTPTSPSSPGLSPVPPPDKVDGFSRRSLR      37
gi|142364854:598-2091              -----------------------METKLPPASTPTSPSSPGLSPVPPPDKVDGFSRRSLR      37
gb|L42374.1|HUMPP2ABA:326-1819     -----------------------METKLPPASTPTSPSSPGLSPVPPPDKVDGFSRRSLR      37
gi|587003404:628-2121              -----------------------METKLPPASTPTSPSSPGLSPVPPPDKVDGFSRRSLR      37
gi|641793336:1879-3372             -----------------------METKLPPAGTPTSPSSPGMSPVPPPDKVDGFSRRSLR      37
gi|557320144:379-1860              -----------------------MEMKLTPAGTPPSPPSPGLSPVPPPEKVDGFSRRSLR      37
gi|51491868:454-1860               -------------------------------------MSSAATTAPSVDKVDGFSRKSVR      23
gi|657554348:108-1517              -----------------------------------MAMSSAATTSPSVDKVDGFSRKSVR      25
gi|641761822:526-1914              -------------------------------------MSSAPTTPPSVDKVDGFSRKSVR      23
gi|557266651:182-1570              -------------------------------------MSSAPTTPPSVDKVDGFSRKSVR      23
gi|586990135:195-1598              -------------------------------------MSSAPTTPPSVDKVDGFSRKSVR      23
gi|532164714:591-1994              -------------------------------------MSSAPTTPPSVDKVDGFSRKSVR      23
```

```
gi|134085748:529-1932               -------------------------------------MSSAPTTPPSVDKVDGFSRKSVR    23
gi|426233507:51-1454                -------------------------------------MSSAPTTPPSVDKVDGFSRKSVR    23
gi|55741699:574-1977                -------------------------------------MSSAPTTPPSVDKVDGFSRKSVR    23
gi|672056393:586-1989               -------------------------------------MSSAPTTPPSVDKVDGFSRKSVR    23
gi|528511094:406-1860               -----------------------------------------MSAISASEKVDGFTRKSVR    19
gi|657570088:928-2469               -----------------------------------------MSAISASEKVDGFTRKSMR    19
gi|557292702:1-1446                 ------------------------------------MEVTDGKGKIRAPEVKKKKRQDKG    24
gi|641796061:534-1967               ----------------------------------------MSAAISASEKVDGFTRKSVR    20
gi|118130061:626-2086               ------------------------------MSSPSPPAPVACAAISASEKVDGFTRKSVR    30
gi|157823929:178-1638               ------------------------------MSSPSPPAPVACAAISASEKVDGFTRKSVR    30
gi|126165211:499-1968               ---------------------------MSSSSPPPPPAGAASAAISAAEKVDGFTRKSVR    33
gi|426240097:195-1664               ---------------------------MSSSSPPPPPAGAASAAISAAEKVDGFTRKSVR    33
gb|L42373.1|HUMPP2A:572-2032        ------------------------------MSSSSPPAGAASAAISASEKVDGFTRKSVR    30
gi|755806859:84-1547                -----------------------------MSSSSPPAGAANAAAISASEKVDGFTRKSVR    31
                                                                                      :

gi|530584065:87-1661                ------SRMVVDAANSN----GPFQPVALLHIRDVPPAEQEKLFIQKLRQCCVLFDFVSD    58
gi|557265256:74-1648                ------SRMVVDAANSN----GPFQPVALLHIRDVPPADQEKLFIQKLRQCCVLFDFVSD    58
gi|755745075:318-1892               ------SRMVVDAANSN----GPFQPVALLHIRDVPPADQEKLFIQKLRQCCVLFDFVSD    58
gi|555290051:121-1695               ------SGMVVDAASSN----GPFQPVALLHIRDVPPADQEKLFIQKLRQCCVLFDFVSD    58
gi|300798097:82-1656                ------SGMVVDAASSN----GPFQPVALLHIRDVPPADQEKLFIQKLRQCCVLFDFVSD    58
gi|240849335:141-1715               ------SRMVVDAANSN----GPFQPVVLLHIRDVPPADQEKLFIQKLRQCCVLFDFVSD    58
gi|803180317:2708-4297              ------SRMVVDAANSN----GPFQPVALLHIRDVPPADQEKLFIQKLRQCCVLFDFVSD    58
gi|134085945:20-1594                ------SRMVVDAASSN----GPFQPVALLHIRDVPPADQEKLFIQKLRQCCVLFDFVSD    58
gi|688603875:111-1688               ---KDEARMVLDAPSAN----GPFQPVALMHFRDVPPAEQEKLFVQKLRQCCVLFDFVSD    58
gi|657585651:111-1688               -------GMVLDALSSN----GPFQPVALMHFRDVPPAEQEKLFIQKLRQCCVLFDFVSD    58
gi|657585641:75-1811                TPVKKDKRQSSSRFSLS----NNRELQKLPAFKDVPPAEQEKLFIQKLRQCCVLFDFVSD    111
gi|50726891:63-1868                 QIVKKERRQSSSRFNLS----KNRELQKLPALKDAPPIDREELFVQKLRQCCVLFDFVTD    121
gi|426251100:1-1800                 QIVKKERRQSSSRFNLS----KNRELQKLPALKDSPTQEREELFIQKLRQCCVLFDFVSD    130
gi|14669809:89-1873                 QIVKKERRQSSFPFNLN----KNRELQKLPALKDSPTQEREELFIQKLRQCCVLFDFVSD    126
gi|672023745:791-2578               QIVKKERRQSSSRFNLS----KNRELQKLPALKDSPTQEREELFIQKLRQCCVLFDFVSD    127
gb|L76702.1|HUMB56DA:188-1996       QIVKKERRQSSSRFNLS----KNRELQKLPALKDSPTQEREELFIQKLRQCCVLFDFVSD    134
gi|329744635:107-1909               QIVKKERRQSSSRFNLS----KNRELQKLPALKDSPTQEREELFIQKLRQCCVLFDFVSD    132
gi|755728309:140-1948               QIVKKERRQSSSRFNLS----KNRELQKLPALKDSPTQEREELFIQKLRQCCVLFDFVSD    134
gi|641758569:96-1892                QIVKKERRHSSSRFNLT----KNRELQKLPALKDAPAHEREELFIQKLRQCCVLFDFISD    130
gi|557295947:96-1958                QIVKKERRHSSSRFNLS----KNRELQKLPALKDAPPHEREELFIQKLRQCCVLFDFISD    152
gi|688564053:1400-2911              RAR-QRRSHSSSQFRYQSS---QVELTPLPLLKDAPVAELHDLFCKKLQQCCVLFDFL-D    91
gi|657563479:1-1563                 RAR-QRRSHSSSQFRYQSS---QVELTPLPLLKDAPVAELHDLFCKKLQQCCVLFDFL-D    90
gi|115497699:359-1849               RAR-PRRSHSSSQFRYQSN---QQELTPLPLLKDVPASELHELLSRKLAQCGVMFDFL-D    92
gi|803191297:577-2067               RAR-PRRSHSSSQFRXQSN---QQELTPLPLLKDVPASELHELLSRKLAQCGVMFDFL-D    92
gi|60593006:48-1541                 RAR-PRRSHSSSQFRYQSN---QQELTPLPLLKDVPASELHELLSRKLAQCGVMFDFL-D    92
gi|142364854:598-2091               RAR-PRRSHSSSQFRYQSN---QQELTPLPLLKDVPASELHELLSRKLAQCGVMFDFL-D    92
gb|L42374.1|HUMPP2ABA:326-1819      RAR-PRRSHSSSQFRYQSN---QQELTPLPLLKDVPASELHELLSRKLAQCGVMFDFL-D    92
gi|587003404:628-2121               RAR-PRRSHSSSQFRYQSN---QQELTPLPLLKDVPASELHELLSRKLAQCGVMFDFL-D    92
gi|641793336:1879-3372              RSR-QRRSHSSSQFRYQSN---QQELTPLPLLKDVAVAELHELLCKKLQQCCVLFDFL-D    92
gi|557320144:379-1860               RTR-PRRSHSSSQFRYQSS---QQELTPLPLLKDVAVSELHELLGRKLQQCCVLFDFL-D    92
```

```
gi|51491868:454-1860                KAR-QKRAQSSSQFRSQDK---PIELVALPLLKDVSAQEQPELFLKKLQQCCTLFDFM-D    78
gi|657554348:108-1517               KAK-QKRSQSSSQFRSQGK---PIELTPLPLLKDVPAPEQPELFLKKLQQCCTVFDFM-D    80
gi|641761822:526-1914               KAR-QKRSQSSSQFRSQGK---PIELTPLPLLKDVPSSEQPELFLKKLQQCCVIFDFM-D    78
gi|557266651:182-1570               KAR-QKRSQSSSQFRSQGK---PIELTPLPLLKDVPSSEQPELFLKKLQQCCVIFDFM-D    78
gi|586990135:195-1598               KAR-QKRSQSSSQFRSQGK---PIELTPLPLLKDVPSSEQPELFLKKLQQCCVIFDFM-D    78
gi|532164714:591-1994               KAR-QKRSQSSSQFRSQGK---PIELTPLPLLKDVPSSEQPELFLKKLQQCCVIFDFM-D    78
gi|134085748:529-1932               KAR-QKRSQSSSQFRSQGK---PIELTPLPLLKDVPSSEQPELFLKKLQQCCVIFDFM-D    78
gi|426233507:51-1454                KAR-QKRSQSSSQFRSQGK---PIELTPLPLLKDVPSSEQPELFLKKLQQCCVIFDFM-D    78
gi|55741699:574-1977                KAR-QKRSQSSSQFRSQGK---PIELTPLPLLKDVPTSEQPELFLKKLQQCCVIFDFM-D    78
gi|672056393:586-1989               KAR-QKRSQSSSQFRSQGK---PIELTPLPLLKDVPTSEQPELFLKKLQQCCVIFDFM-D    78
gi|528511094:406-1860               KAQRQRVCQGSSQFAVNSSRPAAPEISALPQLKDASSTEQHELFMQKLQQCCKLFDFY-D    78
gi|657570088:928-2469               KAQKQRKSQGSSQYRTQSA---PVELSPLPQLKDAPSTEQQELFTQKLQQCCMLFDFL-D    75
gi|557292702:1-1446                 RAKMRKRSQGSSQFRSQSS---QVELSPLPQLKDATSNEQQELFCQKLQQCCILFDFI-D    80
gi|641796061:534-1967               KAQRQKRSQGSSQFRSQSS---QVELSPLPQLKDATSNEQQDLFCQKLQQCCILFDFM-D    76
gi|118130061:626-2086               KAQRQKRSQGSSQFRSQGS---QAELHPLPQLKDATSNEQQELFCQKLQQCCVLFDFM-D    86
gi|157823929:178-1638               KAQRQKRSQGSSQFRSQGS---QAELHPLPQLKDATSNEQQELFCQKLQQCCVLFDFM-D    86
gi|126165211:499-1968               KAQRQKRSQGSSQFRSQCS---QPELHPLPQLKDATSNEQQELFCQKLQQCCILFDFM-D    89
gi|426240097:195-1664               KAQRQKRSQGSSQFRSQCS---QPELHPLPQLKDATSNEQQELFCQKLQQCCILFDFM-D    89
gb|L42373.1|HUMPP2A:572-2032        KAQRQKRSQGSSQFRSQGS---QAELHPLPQLKDATSNEQQELFCQKLQQCCILFDFM-D    86
gi|755806859:84-1547                KAQRQKRSQGSSQFRSQGS---QAELHPLPQLKDATSNEQQELFCQKLQQCCILFDFM-D    87
                                                            :   *  ::*    :  .*: :** **  :***  *

gi|530584065:87-1661                PLSDLKWKEVKRAALSEMVEYITHNRNVITEPIYPE-VVHMFAVNMFRTLPPSSNPTGAE    117
gi|557265256:74-1648                PLSDLKWKEVKRAALSEMVEYITHNRNVITEPIYPE-VVHMFAVNMFRTLPPSSNPTGAE    117
gi|755745075:318-1892               PLSDLKWKEVKRAALSEMVEYITHNRNVITEPIYPE-VVHMFAVNMFRTLPPSSNPTGAE    117
gi|555290051:121-1695               PLSDLKWKEVKRAALSEMVEYITHNRNVITEPIYPE-AVHMFAVNMFRTLPPSSNPTGAE    117
gi|300798097:82-1656                PLSDLKWKEVKRAALSEMVEYITHNRNVITEPIYPE-AVHMFAVNMFRTLPPSSNPTGAE    117
gi|240849335:141-1715               PLSDLKWKEVKRAALSEMVEYITHNRNVITEPIYPE-VVHMFAVNMFRTLPPSSNPTGAE    117
gi|803180317:2708-4297              PLSDLKWKEVKRAALSEMVEYITHNRNVITEPIYPE-VVHMFAVNMFRTLPPSSNPTGAE    117
gi|134085945:20-1594                PLSDLKWKEVKRAALSEMVEYITHNRNVITEPIYPE-VVHMFAVNMFRTLPPSSNPTGAE    117
gi|688603875:111-1688               PLSDLKWKEVKRAALSEMVEYITHNRNVITEPIYPE-VVHMFAVNMFRTLPPSSNPTGAE    117
gi|657585651:111-1688               PLSDLKWKEVKRAALSEMVEYITHNRNVITEPIYPE-VVHMFAVNMFRTLPPSSNPTGAE    117
gi|657585641:75-1811                PLSDLKWKEVKRAALSEMVEYITHNRNVITEPIYPE-VVHMFAVNMFRTLPPSSNPTGAE    170
gi|50726891:63-1868                 PLSDLKYKEVKRAGLNEMVEYITHNRDVVTESIYPE-AVIMFSVNLFRTLPPSSNPTGAE    180
gi|426251100:1-1800                 PLSDLKFKEVKRAGLNEMVEYITHSRDVVTEAIYGPGCTPQFSVNLFRTLPPSSNPTGAE    190
gi|14669809:89-1873                 PLSDLKCKEVKRAGLNEMVEYITHSRDVVTEAIYPEA-VTMFSVNLFRTLPPSSNPTGAE    185
gi|672023745:791-2578               PLSDLKFKEVKRAGLNEMVEYITHSRDVVTEAIYPEA-VTMFSVNLFRTLPPSSNPTGAE    186
gb|L76702.1|HUMB56DA:188-1996       PLSDLKFKEVKRAGLNEMVEYITHSRDVVTEAIYPEA-VTMFSVNLFRTLPPSSNPTGAE    193
gi|329744635:107-1909               PLSDLKFKEVKRAGLNEMVEYITHSRDVVTEAIYPEA-VTMFSVNLFRTLPPSSNPTGAE    191
gi|755728309:140-1948               PLSDLKFKEVKRAGLNEMVEYITHSRDVVTEAIYPEA-VTMFSVNLFRTLPPSSNPTGAE    193
gi|641758569:96-1892                PLSDLKFKEVKRAGLNEMVEYITHNRDVVTEAIYPE-AVIMFSVNLFRTLPPSSNPTGAE    189
gi|557295947:96-1958                PLSDLKFKEVKRAGLNEMVEYITHNRDVITEAIYPE-AVIMFSVNLFRTLPPSSNPTGAE    211
gi|688564053:1400-2911              CVADLKGKEIKRAALNELVESVATSRGVLIEPLYPE-AIKMISVNIFRTLPPSENP---E    147
gi|657563479:1-1563                 CVADLKGKEIKRAALNELVESVATSRGVLIEPLYPE-AIKMISVNIFRTLPPSENP---E    146
gi|115497699:359-1849               CVADLKGKEVKRAALNELVECVGSTRGVLIEPVYPD-IIRMISVNIFRTLPPSENP---E    148
gi|803191297:577-2067               CVADLKGKEVKRAALNELVECVGSTRGVLIEPVYPD-IIRMISVNIFRTLPPSENP---E    148
```

```
gi|60593006:48-1541                 CVADLKGKEVKRAALNELVECVGSTRGVLIEPVYPD-IIRMISVNIFRTLPPSENP---E      148
gi|142364854:598-2091               CVADLKGKEVKRAALNELVECVGCTRGVLIEPVYPD-IIRMISVNIFRTLPPSENP---E      148
gb|L42374.1|HUMPP2ABA:326-1819      CVADLKGKEVKRAALNELVECVGSTRGVLIEPVYPD-IIRMISVNIFRTLPPSENP---E      148
gi|587003404:628-2121               CVADLKGKEVKRAALNELVECVGSTRGVLIEPVYPD-IIRMISVNIFRTLPPSENP---E      148
gi|641793336:1879-3372              CVADLKGKEIKRAALNELVECVATNRGVLIEPVYPE-IIKMISVNIFRTLPPTENP---E      148
gi|557320144:379-1860               PVADLKGKEVKRAALAELVECVATARGLLIEPTYPE-AVRMISVNIFRTLPPSENP---E      148
gi|51491868:454-1860                TLSDLKMKEYKRSTLNELVDYVTLSRGYLTEQTYPE-VVKMVSYNIFRTLPPSDSN---E      134
gi|657554348:108-1517               TLSDLKMKEYKRSTLNELVDYVTVSRGYLTEQAYPE-VVKMVSHNIFRTLPPSDSN---E      136
gi|641761822:526-1914               TLSDLKMKEYKRSTLNELVDYITISRGCLTEQTYPE-VVRMVSCNIFRTLPPSDSN---E      134
gi|557266651:182-1570               TLSDLKMKEYKRSTLNELVDYITISRGCLTEQTYPE-VVRMVSCNIFRTLPPSDSN---E      134
gi|586990135:195-1598               TLSDLKMKEYKRSTLNELVDYITISRGCLTEQTYPE-VVRMVSCNIFRTLPPSDSN---E      134
gi|532164714:591-1994               TLSDLKMKEYKRSTLNELVDYITISRGCLTEQTYPE-VVRMVSCNIFRTLPPSDSN---E      134
gi|134085748:529-1932               TLSDLKMKEYKRSTLNELVDYITISRGCLTEQTYPE-VVRMVSCNIFRTLPPSDSN---E      134
gi|426233507:51-1454                TLSDLKMKEYKRSTLNELVDYITISRGCLTEQTYPE-VVRMVSCNIFRTLPPSDSN---E      134
gi|55741699:574-1977                TLSDLKMKEYKRSTLNELVDYITISRGCLTEQTYPE-VVRMVSCNIFRTLPPSDSN---E      134
gi|672056393:586-1989               TLSDLKMKEYKRSTLNELVDYITISRGCLTEQTYPE-VVRMVSCNIFRTLPPSDSN---E      134
gi|528511094:406-1860               TVTDLKSKETKRATLSELVDYVSTNRGVLVEPVYPE-ITTMVSTNIFRTLPPSENP---D      134
gi|657570088:928-2469               SVTDLKSKEIKRATLNELVDFVSTNRGVLVESAYPE-ITNMISTNIFRALPPSDNP---D      131
gi|557292702:1-1446                 SVSDLKSKEIKRATLNELVEYVSTNRGVLVEPAYAD-IVKMISSNIFRTLPPSDNP---D      136
gi|641796061:534-1967               SVSDLKSKEIKRATLNELVEYVSTTRGVIVESAYAD-IVKMISSNIFRTLPPSDNP---D      132
gi|118130061:626-2086               SVSDLKSKEIKRATLNELVEYVSTNRGVIVESAYSD-IVKMISANIFRTLPPSDNP---D      142
gi|157823929:178-1638               SVSDLKSKEIKRATLNELVEYVSTNRGVIVESAYSD-IVKMISANIFRTLPPSDNP---D      142
gi|126165211:499-1968               SVSDLKSKEIKRATLNELVEYVSTNRGVIVESAYSD-IVKMISANIFRTLPPSDNP---D      145
gi|426240097:195-1664               SVSDLKSKEIKRATLNELVEYVSTNRGVIVESAYSD-IVKMISANIFRTLPPSDNP---D      145
gb|L42373.1|HUMPP2A:572-2032        SVSDLKSKEIKRATLNELVEYVSTNRGVIVESAYSD-IVKMISANIFRTLPPSDNP---D      142
gi|755806859:84-1547                SVSDLKSKEIKRATLNELVEYVSTNRGVIVESAYSD-IVKMISANIFRTLPPSDNP---D      143
                                     ::*** ** **: * *:*: :   *  : *  *       .: *:**:***:..    :

gi|530584065:87-1661                FDPEEDEPTLEAAWPHLQLVYEFFLRFLESPDFQPNVAKKYIDQKFVLQLLELFDSEDPR      177
gi|557265256:74-1648                FDPEEDEPTLEAAWPHLQLVYEFFLRFLESPDFQPNIAKKYIDQKFVLQLLELFDSEDPR      177
gi|755745075:318-1892               FDPEEDEPTLEAAWPHLQLVYEFFLRFLESPDFQPNIAKKYIDQKFVLQLLELFDSEDPR      177
gi|555290051:121-1695               FDPEEDEPTLEAAWPHLQLVYEFFLRFLESPDFQPNIAKKYIDQKFVLQLLELFDSEDPR      177
gi|300798097:82-1656                FDPEEDEPTLEAAWPHLQLVYEFFLRFLESPDFQPNIAKKYIDQKFVLQLLELFDSEDPR      177
gi|240849335:141-1715               FDPEEDEPTLEAAWPHLQLVYEFFLRFLESPDFQPNIAKKYIDQKFVLQLLELFDSEDPR      177
gi|803180317:2708-4297              FDPEEDEPTLEAAWPHLQLVYEFFLRLLESPDFQPNIAKKYIDQKFVLQLLELFDSEDPR      177
gi|134085945:20-1594                FDPEEDEPTLEAAWPHLQLVYEFFLRLLESPDFQPNIAKKYIDQKFVLQLLELFDSEDPR      177
gi|688603875:111-1688               FDPEEDEPTLEAAWPHLQLVYEFFLRFLESPDFQPNIAKKYIDQKFVLQLLELFDSEDPR      177
gi|657585651:111-1688               FDPEEDEPTLEAAWPHLQLVYEFFLRFLESPDFQPNIAKKYIDQKFVMQLLELFDSEDPR      177
gi|657585641:75-1811                FDPEEDEPTLEAAWPHLQLVYEFFLRFLESPDFQPNIAKKYIDQKFVMQLLELFDSEDPR      230
gi|50726891:63-1868                 FDPEEDEPTLEAAWPHLQLVYEFFLRFLESPDFQPNIAKKYIDQKFVLSLLELFDSEDPR      240
gi|426251100:1-1800                 FDPEEDEPTLEAAWPHLQLVYEFFLRFLESPDFQPNIAKKYIDQKFVLALLDLFDSEDPR      250
gi|14669809:89-1873                 FDPEEDEPTLEAAWPHLQLVYEFFLRFLESPDFQPNIAKKYIDQKFVLALLDLFDSEDPR      245
gi|672023745:791-2578               FDPEEDEPTLEAAWPHLQLVYEFFLRFLESPDFQPNIAKKYIDQKFVLALLDLFDSEDPR      246
gb|L76702.1|HUMB56DA:188-1996       FDPEEDEPTLEAAWPHLQLVYEFFLRFLESPDFQPNIAKKYIDQKFVLALLDLFDSEDPR      253
gi|329744635:107-1909               FDPEEDEPTLEAAWPHLQLVYEFFLRFLESPDFQPNIAKKYIDQKFVLALLDLFDSEDPR      251
gi|755728309:140-1948               FDPEEDEPTLEAAWPHLQLVYEFFLRFLESPDFQPNIAKKYIDQKFVLALLDLFDSEDPR      253
```

```
gi|641758569:96-1892             FDPEEDEPTLEAAWPHLQLVYEFFLRFLESPDFQPNIAKKYIDQKFVLSLLDLFDSEDPR	249
gi|557295947:96-1958             FDPEEDEPTLEAAWPHLQLVYEFFLRFLESPDFQPNVAKKYIDQKFVLSLLDLFDSEDPR	271
gi|688564053:1400-2911           FDPEEDEPALEASWPHLQLVYEFFLRFLESPDFQPSLAKRYVDQKFVLQLLELFDSEDPR	207
gi|657563479:1-1563              FDPEEDEPTLEASWPHLQLVYEFFLRFLESPDFQPSMAKRYVDQKFVLQLLELFDSEDPR	206
gi|115497699:359-1849            FDPEEDEPNLEPSWPHLQLVYEFFLRFLESPDFQPSVAKRYVDQKFVLMLLELFDSEDPR	208
gi|803191297:577-2067            FDPEEDEPNLEPSWPHLQLVYEFFLRFLESPDFQPSVAKRYVDQKFVLMLLELFDSEDPR	208
gi|60593006:48-1541              FDPEEDEPNLEPSWPHLQLVYEFFLRFLESPDFQPSVAKRYVDQKFVLMLLELFDSEDPR	208
gi|142364854:598-2091            FDPEEDEPNLEPSWPHLQLVYEFFLRFLESPDFQPSVAKRYVDQKFVLMLLELFDSEDPR	208
gb|L42374.1|HUMPP2ABA:326-1819   FDPEEDEPNLEPSWPHLQLVYEFFLRFLESPDFQPSVAKRYVDQKFVLMLLELFDSEDPR	208
gi|587003404:628-2121            FDPEEDEPNLEPSWPHLQLVYEFFLRFLESPDFQPSVAKRYVDQKFVLMLLELFDSEDPR	208
gi|641793336:1879-3372           FDPEEDEPNLEPSWPHLQLVYEFFLRFLESPDFQPSVAKRYVDQKFVLLLLELFDSEDPR	208
gi|557320144:379-1860            FDPEEDEPNLEPSWPHLQLVYEFFLRFLESPDFQPSVAKRYVDQKFVLMLLELFDSEDPR	208
gi|51491868:454-1860             FDPEEDEPTLEASWPHLQLVYEFFIRFLESQEFQPSAAKKYIDQKFVLQLLELFDSEDPR	194
gi|657554348:108-1517            FDPEEDEPTLEASWPHLQLVYEFFIRFLESQEFQPSIAKKYIDQKFVLQLLELFDSEDPR	196
gi|641761822:526-1914            FDPEEDEPTLEASWPHLQLVYEFFIRFLESQEFQPSIAKKYIDQKFVLQLLELFDSEDPR	194
gi|557266651:182-1570            FDPEEDEPTLEASWPHLQLVYEFFIRFLESQEFQPSIAKKYIDQKFVLQLLELFDSEDPR	194
gi|586990135:195-1598            FDPEEDEPTLEASWPHLQLVYEFFIRFLESQEFQPSIAKKYIDQKFVLQLLELFDSEDPR	194
gi|532164714:591-1994            FDPEEDEPTLEASWPHLQLVYEFFIRFLESQEFQPSIAKKYIDQKFVLQLLELFDSEDPR	194
gi|134085748:529-1932            FDPEEDEPTLEASWPHLQLVYEFFIRFLESQEFQPSIAKKYIDQKFVLQLLELFDSEDPR	194
gi|426233507:51-1454             FDPEEDEPTLEASWPHLQLVYEFFIRFLESQEFQPSIAKKYIDQKFVLQLLELFDSEDPR	194
gi|55741699:574-1977             FDPEEDEPTLEASWPHLQLVYEFFIRFLESQEFQPSIAKKYIDQKFVLQLLELFDSEDPR	194
gi|672056393:586-1989            FDPEEDEPTLEASWPHLQLVYEFFIRFLESQEFQPSIAKKYIDQKFVLQLLELFDSEDPR	194
gi|528511094:406-1860            FDPEEDEPTLEASWPHMQLVYEFFLRFLENPDFQPSTAKRYIDQKFVLQLLELFDSEDPR	194
gi|657570088:928-2469            FDPEEDEPTLEASWPHMQLVYEFLLRFLENPDFQPSIAKRHIDQKFVLQLLELFDSEDPR	191
gi|557292702:1-1446              FDPEEDEPTLEASWPHIQLVYEFFLRFLESLDFQPSIAKRYIDQKFVQQLLELFDSEDPR	196
gi|641796061:534-1967            FDPEEDEPTLEASWPHIQLVYEFFLRFLESPDFQPSIAKRYIDQKFVQQLLELFDSEDPR	192
gi|118130061:626-2086            FDPEEDEPTLEASWPHIQLVYEFFLRFLESPDFQPSIAKRYIDQKFVQQLLELFDSEDPR	202
gi|157823929:178-1638            FDPEEDEPTLEASWPHIQLVYEFFLRFLESPDFQPSIAKRYIDQKFVQQLLELFDSEDPR	202
gi|126165211:499-1968            FDPEEDEPTLEASWPHIQLVYEFFLRFLESPDFQPSIAKRYIDQKFVQQLLELFDSEDPR	205
gi|426240097:195-1664            FDPEEDEPTLEASWPHIQLVYEFFLRFLESPDFQPSIAKRYIDQKFVQQLLELFDSEDPR	205
gb|L42373.1|HUMPP2A:572-2032     FDPEEDEPTLEASWPHIQLVYEFFLRFLESPDFQPSIAKRYIDQKFVQQLLELFDSEDPR	202
gi|755806859:84-1547             FDPEEDEPTLEASWPHIQLVYEFFLRFLESPDFQPSIAKRYIDQKFVQQLLELFDSEDPR	203
                                 ******** ** :***:******::*:**. :***. **:::*****  **:********

gi|530584065:87-1661             ERDFLKTTLHRIYGKFLGLRAYIRKQINNIFYRFIYETEHHNGIAELLEILGSIINGFAL	237
gi|557265256:74-1648             ERDFLKTTLHRIYGKFLGLRAYIRKQINNIFYRFIYETEHHNGIAELLEILGSIINGFAL	237
gi|755745075:318-1892            ERDFLKTTLHRIYGKFLGLRAYIRKQINNIFYRFIYETEHHNGIAELLEILGSIINGFAL	237
gi|555290051:121-1695            ERDFLKTTLHRIYGKFLGLRAYIRKQINNIFYRFIYETEHHNGIAELLEILGSIINGFAL	237
gi|300798097:82-1656             ERDFLKTTLHRIYGKFLGLRAYIRKQINNIFYRFIYETEHHNGIAELLEILGSIINGFAL	237
gi|240849335:141-1715            ERDFLKTTLHRIYGKFLGLRAYIRKQINNIFYRFIYETEHHNGIAELLEILGSIINGFAL	237
gi|803180317:2708-4297           ERDFLKTTLHRIYGKFLGLRAYIRKQINNIFYRFIYETEHHNGIAELLEILGSIINGFAL	237
gi|134085945:20-1594             ERDFLKTTLHRIYGKFLGLRAYIRKQINNIFYRFIYETEHHNGIAELLEILGSIINGFAL	237
gi|688603875:111-1688            ERDFLKTTLHRIYGKFLGLRAYIRKQINNIFYRFIYETEHHNGIAELLEILGSIINGFAL	237
gi|657585651:111-1688            ERDFLKTTLHRIYGKFLGLRAYIRKQINNIFYRFIYETEHHNGIAELLEILGSIINGFAL	237
gi|657585641:75-1811             ERDFLKTTLHRIYGKFLGLRAYIRKQINNIFYRFIYETEHHNGIAELLEILGSIINGFAL	290
gi|50726891:63-1868              ERDFLKTILHRIYGKFLGLRAYIRRQINNIFYRFIYETEHHNGIAELLEILGSIINGFAL	300
```

```
gi|426251100:1-1800            ERDFLKTILHRIYGKFLGLRAYIRRQINHIFYRFIYETEHHNGIAELLEILGSIINGFAL    310
gi|14669809:89-1873            ERDFLKTILHRIYGKFLGLRAYIRRQINHIFYRFIYETEHHNGIAELLEILGSIINGFAL    305
gi|672023745:791-2578          ERDFLKTILHRIYGKFLGLRAYIRRQINHIFYRFIYETEHHNGIAELLEILGSIINGFAL    306
gb|L76702.1|HUMB56DA:188-1996  ERDFLKTILHRIYGKFLGLRAYIRRQINHIFYRFIYETEHHNGIAELLEILGSIINGFAL    313
gi|329744635:107-1909          ERDFLKTILHRIYGKFLGLRAYIRRQINHIFYRFIYETEHHNGIAELLEILGSIINGFAL    311
gi|755728309:140-1948          ERDFLKTILHRIYGKFLGLRAYIRRQINHIFYRFIYETEHHNGIAELLEILGSIINGFAL    313
gi|641758569:96-1892           ERDFLKTILHRIYGKFLGLRAYVRRQINNIFYRFIYETEHHNGIAELLEILGSIINGFAL    309
gi|557295947:96-1958           ERDFLKTILHRIYGKFLGLRAYVRRQINNIFYRFIYETEHHNGIAELLEILGSIINGFAL    331
gi|688564053:1400-2911         EREYLKTILHRVYGKLLGLRAYIRKQINNIFLRFIYETERFNGVAELLEILGSIINGFAL    267
gi|657563479:1-1563            EREYLKTILHRVYGKLLGLRAYIRKQINNIFLRFIYETEHFNGVAELLEILGSIINGFAL    266
gi|115497699:359-1849          EREYLKTILHRVYGKFLGLRAYIRKQCSHIFLRFIYEFEHFNGVAELLEILGSIINGFAL    268
gi|803191297:577-2067          EREYLKTILHRVYGKFLGLRAYIRKQCSHIFLRFIYEFEHFNGIAELLEILGSIINGFAL    268
gi|60593006:48-1541            EREYLKTILHRVYGKFLGLRAYIRKQCNHIFLRFIYELEHFNGVAELLEILGSIINGFAL    268
gi|142364854:598-2091          EREYLKTILHRVYGKFLGLRAYIRKQCNHIFLRFIYELEHFNGVAELLEILGSIINGFAL    268
gb|L42374.1|HUMPP2ABA:326-1819 EREYLKTILHRVYGKFLGLRAYIRKQCNHIFLRFIYEFEHFNGVAELLEILGSIINGFAL    268
gi|587003404:628-2121          EREYLKTILHRVYGKFLGLRAYIRKQCSHIFLRFIYELEHFNGVAELLEILGSIINGFAL    268
gi|641793336:1879-3372         EREYLKTILHRVYGKFLGLRAYIRKQCNNIFLRFIYETEHFNGVAELLEILGSIINGFAL    268
gi|557320144:379-1860          EREYLKTILHRVYGKFLGLRAYIRKQCNNIFLRFIYETEHFNGVAELLEILGSIINGFAL    268
gi|51491868:454-1860           ERDCLKTVLHRIYGKFLGLRAFIRKQINNIFLCFVYETERFNGVAELLEILGSIINGFAL    254
gi|657554348:108-1517          ERDYLKTVLHRIYGKFLGLRAFIRKQINNIFLRFVYETEHFNGVAELLEILGSIINGFAL    256
gi|641761822:526-1914          ERDYLKTVLHRIYGKFLGLRAFIRKQINNIFLRFVYETEHFNGVAELLEILGSIINGFAL    254
gi|557266651:182-1570          ERDYLKTVLHRIYGKFLGLRAFIRKQINNIFLRFVYETEHFNGVAELLEILGSIINGFAL    254
gi|586990135:195-1598          ERDYLKTVLHRIYGKFLGLRAFIRKQINNIFLRFVYETEHFNGVAELLEILGSIINGFAL    254
gi|532164714:591-1994          ERDYLKTVLHRIYGKFLGLRAFIRKQINNIFLRFVYETEHFNGVAELLEILGSIINGFAL    254
gi|134085748:529-1932          ERDYLKTVLHRIYGKFLGLRAFIRKQINNIFLRFVYETEHFNGVAELLEILGSIINGFAL    254
gi|426233507:51-1454           ERDYLKTVLHRIYGKFLGLRAFIRKQINNIFLRFVYETEHFNGVAELLEILGSIINGFAL    254
gi|55741699:574-1977           ERDYLKTVLHRIYGKFLGLRAFIRKQINNIFLRFVYETEHFNGVAELLEILGSIINGFAL    254
gi|672056393:586-1989          ERDYLKTVLHRIYGKFLGLRAFIRKQINNIFLRFVYETEHFNGVAELLEILGSIINGFAL    254
gi|528511094:406-1860          EREFLKTILHRIYGKFLGLRAFIRKQINNIFLRFIYETEHFNGVAELLEILGSIINGFAL    254
gi|657570088:928-2469          ERDFLKTILHRIYGKFLGLRAFIRKQINNIFLRFIYETEHFNGVAELLEILGSIINGFAL    251
gi|557292702:1-1446            ERDFLKTVLHRIYGKFLGLRAFIRKQINNIFLRFIYETEHFNGVAELLEILGSIINGFAL    256
gi|641796061:534-1967          ERDFLKTVLHRIYGKFLGLRAFIRKQINNIFLRFIYETEHFNGVAELLEILGSIINGFAL    252
gi|118130061:626-2086          ERDFLKTVLHRIYGKFLGLRAFIRKQINNIFLRFIYETEHFNGVAELLEILGSIINGFAL    262
gi|157823929:178-1638          ERDFLKTVLHRIYGKFLGMRAFIRKQTNNIFLRFIYETEHFNGVAELLEILGSIINGFAL    262
gi|126165211:499-1968          ERDFLKTVLHRIYGKFLGLRAFIRKQINNIFLRFIYETEHFNGVAELLEILGSIINGFAL    265
gi|426240097:195-1664          ERDFLKTVLHRIYGKFLGLRAFIRKQINNIFLRFIYETEHFNGVAELLEILGSIINGFAL    265
gb|L42373.1|HUMPP2A:572-2032   ERDFLKTVLHRIYGKFLGLRAFIRKQINNIFLRFIYETEHFNGVAELLEILGSIINGFAL    262
gi|755806859:84-1547           ERDFLKTVLHRIYGKFLGLRAFIRKQINNIFLRFIYETEHFNGVAELLEILGSIINGFAL    263
                               **: *** ***:***:**:**::*:* ..**  *:** *:.**:****************

gi|530584065:87-1661           PLKEEHKIFLLKVLLPLHKVKSLSVYHPQLAYCVVQFLEKDSTLTEPVVMALLKYWPKTH    297
gi|557265256:74-1648           PLKEEHKIFLLKVLLPLHKVKSLSVYHPQLAYCVVQFLEKDSTLTEPVVMALLKYWPKTH    297
gi|755745075:318-1892          PLKEEHKIFLLKVLLPLHKVKSLSVYHPQLAYCVVQFLEKDSTLTEPVVMALLKYWPKTH    297
gi|555290051:121-1695          PLKEEHKIFLLKVLLPLHKVKSLSVYHPQLAYCVVQFLEKDSTLTEPVVMALLKYWPKTH    297
gi|300798097:82-1656           PLKEEHKIFLLKVLLPLHKVKSLSVYHPQLAYCVVQFLEKDSTLTEPVVMALLKYWPKTH    297
gi|240849335:141-1715          PLKEEHKIFLLKVLLPLHKVKSLSVYHPQLAYCVVQFLEKDSTLTEPVVMALLKYWPKTH    297
```

```
gi|803180317:2708-4297          PLKEEHKIFLLKVLLPLHKVKSLSVYHPQLAYCVVQFLEKDSTLTEPVVMALLKYWPKTH     297
gi|134085945:20-1594            PLKEEHKIFLLKVLLPLHKVKSLSVYHPQLAYCVVQFLEKDSTLTEPVVMALLKYWPKTH     297
gi|688603875:111-1688           PLKEEHKIFLLKVLLPLHKVKSLSVYHPQLAYCVVQFLEKDSTLTEPVVMALLKYWPKTH     297
gi|657585651:111-1688           PLKEEHKIFLLKVLLPLHKVKSLSVYHPQLAYCVVQFLEKDSTLTEPTVMALLKYWPKTH     297
gi|657585641:75-1811            PLKEEHKIFLLKVLLPLHKVKSLSVYHPQLAYCVVQFLEKDSTLTEPTVMALLKYWPKTH     350
gi|50726891:63-1868             PLKEEHKMFLIRVLLPLHKVKSLSVYHPQLAYCVVQFLEKDSSLTEPVIMGLLKFWPKTH     360
gi|426251100:1-1800             PLKEEHKMFLIRVLLPLHKVKSLSVYHPQLAYCVVQFLEKESSLTEPVIVGLLKFWPKTH     370
gi|14669809:89-1873             PLKEEHKVFLVRVLLPLHKVKSLSVYHPQLAYCVVQFLEKESSLTEPVIVGLLKFWPKTH     365
gi|672023745:791-2578           PLKEEHKMFLIRVLLPLHKVKSLSVYHPQLAYCVVQFLEKESSLTEPVIVGLLKFWPKTH     366
gb|L76702.1|HUMB56DA:188-1996   PLKEEHKMFLIRVLLPLHKVKSLSVYHPQLAYCVVQFLEKESSLTEPVIVGLLKFWPKTH     373
gi|329744635:107-1909           PLKEEHKMFLIRVLLPLHKVKSLSVYHPQLAYCVVQFLEKESSLTEPVIVGLLKFWPKTH     371
gi|755728309:140-1948           PLKEEHKMFLIRVLLPLHKVKSLSVYHPQLAYCVVQFLEKESSLTEPVIVGLLKFWPKTH     373
gi|641758569:96-1892            PLKEEHKMFLIRVLLPLHKVKSLSVYHPQLAYCVVQFLEKESSLTEPVIVGLLKFWPKTH     369
gi|557295947:96-1958            PLKEEHKMFLIRVLLPLHKVKSLSVYHPQLAYCVVQFLEKDSSLTEPVIMGLLKFWPKTH     391
gi|688564053:1400-2911          PLKSEHKQFLVRVLIPLHTAKSLSIFHAQLAYCVVQFMEKDATVTEHIIRGLLRYWPKTC     327
gi|657563479:1-1563             PLKAEHKQFLVRVLIPLHTAKSLSIFHAQLAYCVVQFMEKDATVTEYIIRGLLKYWPKTC     326
gi|115497699:359-1849           PLKTEHKQFLVRVLIPLHSVKSLSVFHAQLAYCVVQFLEKDATLTEHVIRGLLKYWPKTC     328
gi|803191297:577-2067           PLKTEHKQFLVRVLIPLHSVKSLSVFHAQLAYCVVQFLEKDATLTEHVIRGLLKYWPKTC     328
gi|60593006:48-1541             PLKTEHKQFLVRVLIPLHSVKSLSVFHAQLAYCVVQFLEKDATLTEHVIRGLLKYWPKTC     328
gi|142364854:598-2091           PLKTEHKQFLVRVLIPLHSVKSLSVFHAQLAYCVVQFLEKDATLTEHVIRGLLKYWPKTC     328
gb|L42374.1|HUMPP2ABA:326-1819  PLKTEHKQFLVRVLIPLHSVKSLSVFHAQLAYCVVQFLEKDATLTEHVIRGLLKYWPKTC     328
gi|587003404:628-2121           PLKTEHKQFLVRVLIPLHSVKSLSVFHAQLAYCVVQFLEKDATLTEHVIRGLLKYWPKTC     328
gi|641793336:1879-3372          PLKTEHKHFLVRVLIPLHSVKSLSIFHAQLAYCVVQFLEKDATLTEHVIRGLLKYWPKTC     328
gi|557320144:379-1860           PLKAEHKQFLVRVLIPLHSVKALSVFHAQLAYCVVQFLEKDATLTEHVIRGLLKYWPKTC     328
gi|51491868:454-1860            PLKAEHKQFLVKVLLPLHTVRSLSLFHAQLAYCIVQFLEKDPTLTEPVIRGLLKFWPKTC     314
gi|657554348:108-1517           PLKAEHKQFLVKVLIPLHTVRSLSLFHAQLAYCIVQFLEKDPTLTEPVIRGLLKFWPKTC     316
gi|641761822:526-1914           PLKAEHKQFLVKVLIPLHTVRSLSLFHAQLAYCIVQFLEKDPSLTEPVIRGLMKFWPKTC     314
gi|557266651:182-1570           PLKAEHKQFLVKVLIPLHTVRSLSLFHAQLAYCIVQFLEKDPSLTEPVIRGLMKFWPKTC     314
gi|586990135:195-1598           PLKAEHKQFLVKVLIPLHTVRSLSLFHAQLAYCIVQFLEKDPSLTEPVIRGLMKFWPKTC     314
gi|532164714:591-1994           PLKAEHKQFLVKVLIPLHTVRSLSLFHAQLAYCIVQFLEKDPSLTEPVIRGLMKFWPKTC     314
gi|134085748:529-1932           PLKAEHKQFLVKVLIPLHTVRSLSLFHAQLAYCIVQFLEKDPSLTEPVIRGLMKFWPKTC     314
gi|426233507:51-1454            PLKAEHKQFLVKVLIPLHTVRSLSLFHAQLAYCIVQFLEKDPSLTEPVIRGLMKFWPKTC     314
gi|55741699:574-1977            PLKAEHKQFLVKVLIPLHTVRSLSLFHAQLAYCIVQFLEKDPSLTEPVIRGLMKFWPKTC     314
gi|672056393:586-1989           PLKAEHKQFLVKVLIPLHTVRSLSLFHAQLAYCIVQFLEKDPSLTEPVIRGLMKFWPKTC     314
gi|528511094:406-1860           PLKAEHKQFLMKILIPLHTAKPLALFHAQLAYCVVQFLEKDPTLTEVVVRGLLKFWPKTC     314
gi|657570088:928-2469           PLKAEHKQFLMKVLIPLHTAKGLALFHAQLAYCVVQFLEKDPTLTEPVIRGLLKFWPKTC     311
gi|557292702:1-1446             PLKAEHKQFLMKVLIPMHTAKGLALFHAQLAYCVVQFLEKETTLTEPVIRGLLKFWPKTC     316
gi|641796061:534-1967           PLKAEHKQFLMKVLIPMHTAKGLALFHAQLAYCVVQFLEKDTTLTEPVIRGLLKFWPKTC     312
gi|118130061:626-2086           PLKAEHKQFLMKVLIPMHTAKGLALFHAQLAYCVVQFLEKDTTLTEPVIRGLLKFWPKTC     322
gi|157823929:178-1638           PLKAEHKQFLMKVLIPMHTAKGLALFHAQLAYCVVQFLEKDTTLTEPVIRGLLKFWPKTC     322
gi|126165211:499-1968           PLKAEHKQFLMKVLIPMHTAKGLALFHAQLAYCVVQFLEKDTTLTEPVIRGLLKFWPKTC     325
gi|426240097:195-1664           PLKAEHKQFLMKVLIPMHTAKGLALFHAQLAYCVVQFLEKDTTLTEPVIRGLLKFWPKTC     325
gb|L42373.1|HUMPP2A:572-2032    PLKAEHKQFLMKVLIPMHTAKGLALFHAQLAYCVVQFLEKDTTLTEPVIRGLLKFWPKTC     322
gi|755806859:84-1547            PLKAEHKQFLMKVLIPMHTAKGLALFHAQLAYCVVQFLEKDTTLTEPVIRGLLKFWPKTC     323
                                *** *** **:::*:*:*..: *:::* *****:***:**: ::**  : .*:::****
```

```
gi|530584065:87-1661            SPKEVMFLNELEEILDVIEPSEFVKVMEPLFRQLAKCVSSPHFQVAERALYYWNNEYIMS    357
gi|557265256:74-1648            SPKEVMFLNELEEILDVIEPSEFVKVMEPLFRQLAKCVSSPHFQVAERALYYWNNEYIMS    357
gi|755745075:318-1892           SPKEVMFLNELEEILDVIEPSEFVKVMEPLFRQLAKCVSSPHFQVAERALYYWNNEYIMS    357
gi|555290051:121-1695           SPKEVMFLNELEEILDVIEPSEFVKIMEPLFRQLAKCVSSPHFQVAERALYYWNNEYIMS    357
gi|300798097:82-1656            SPKEVMFLNELEEILDVIEPSEFVKIMEPLFRQLAKCVSSPHFQVAERALYYWNNEYIMS    357
gi|240849335:141-1715           SPKEVMFLNELEEILDVIEPSEFVKIMEPLFRQLAKCVSSPHFQVAERALYYWNNEYIMS    357
gi|803180317:2708-4297          SPKEVMFLNELEEILDVIEPSEFVKIMEPLFRQLAKCVSSPHFQVAERALYYWNNEYIMS    357
gi|134085945:20-1594            SPKEVMFLNELEEILDVIEPSEFVKIMEPLFRQLAKCVSSPHFQVAERALYYWNNEYIMS    357
gi|688603875:111-1688           SPKEVMFLNELEEILDVIEPSEFVKVMEPLFRQLAKCVSSPHFQVAERALYYWNNEYIMS    357
gi|657585651:111-1688           SPKEVMFLNELEEILDVIEPSEFVKVMEPLFRQLAKCVSSPHFQVAERALYYWNNEYIMS    357
gi|657585641:75-1811            SPKEVMFLNELEEILDVIEPSEFVKVMEPLFRQLAKCVSSPHFQVAERALYYWNNEYIMS    410
gi|50726891:63-1868             SPKEVMFLNELEEILDVIEPSEFVKVMEPLFRQLAKCVSSPHFQVAERALYYWNNEYIMS    420
gi|426251100:1-1800             SPKEVMFLNELEEILDVIEPSEFSKVMEPLFRQLAKCVSSPHFQVAERALYYWNNEYIMS    430
gi|14669809:89-1873             SPKEVMFLNELEEILDVIEPSEFSKVMEPLFRQLAKCVSSPHFQVAERALYYWNNEYIMS    425
gi|672023745:791-2578           SPKEVMFLNELEEILDVIEPSEFSKVMEPLFRQLAKCVSSPHFQVAERALYYWNNEYIMS    426
gb|L76702.1|HUMB56DA:188-1996   SPKEVMFLNELEEILDVIEPSEFSKVMEPLFRQLAKCVSSPHFQVAERALYYWNNEYIMS    433
gi|329744635:107-1909           SPKEVMFLNELEEILDVIEPSEFSKVMEPLFRQLAKCVSSPHFQVAERALYYWNNEYIMS    431
gi|755728309:140-1948           SPKEVMFLNELEEILDVIEPSEFSKVMEPLFRQLAKCVSSPHFQVAERALYYWNNEYIMS    433
gi|641758569:96-1892            SPKEVMFLNELEEILDVIEPSEFVKVMEPLFRQLAKCVSSPHFQVAERALYYWNNEYIMS    429
gi|557295947:96-1958            SPKEVMFLNELEEILDVIEPSEFVKVMEPLFRQLAKCVSSPHFQVAERALYYWNNEYIMS    451
gi|688564053:1400-2911          TQKEVMFLGEIEEILDVIEPSQFIRVQEPLFKQIAACISSPHFQVAERALYFWNNEYILS    387
gi|657563479:1-1563             TQKEVMFLGEIEEILDVIEPSQFIRVQEPLFKQIAACISSPHFQVAERALYFWNNEYILS    386
gi|115497699:359-1849           TQKEVMFLGEMEEILDVIEPSQFVKIQEPLFKQVARCVSSPHFQVAERALYFWNNEYILS    388
gi|803191297:577-2067           TQKEVMFLGEMEEILDVIEPSQFVKIQEPLFKQVARCVSSPHFQVAERALYFWNNEYILS    388
gi|60593006:48-1541             TQKEVMFLGEMEEILDVIEPSQFVKIQEPLFKQVARCVSSPHFQVAERALYFWNNEYILS    388
gi|142364854:598-2091           TQKEVMFLGEMEEILDVIEPSQFVKIQEPLFKQVARCVSSPHFQVAERALYFWNNEYILS    388
gb|L42374.1|HUMPP2ABA:326-1819  TQKEVMFLGEMEEILDVIEPSQFVKIQEPLFKQVARCVSSPHFQVAERALYFWNNEYILS    388
gi|587003404:628-2121           TQKEVMFLGEMEEILDVIEPSQFVKIQEPLFKQVARCVSSPHFQVAERALYFWNNEYILS    388
gi|641793336:1879-3372          TQKEVMFLGEIEEILDVIEPSQFVKVQEPLFKQIARCISSPHFQVAERALYFWNNEYILS    388
gi|557320144:379-1860           TQKEVMFLGEIEEILDVIEPSQFVKVQQPLFKQVAQCIASPHFQVAERALYFWNNEYILS    388
gi|51491868:454-1860            SQKEVMFLGELEEILDVIEPTQFVKIQELLFKQIARCVSSPHFQVAERALYYWNNEYIMS    374
gi|657554348:108-1517           SQKEVMFLGELEEILDVIEPTQFVKIQEPLFKQISRCVSSPHFQVAERALYYWNNEYIMS    376
gi|641761822:526-1914           SQKEVMFLGELEEILDVIEPSQFVKIQEPLFKQIAKCVSSPHFQVAERALYYWNNEYIMS    374
gi|557266651:182-1570           SQKEVMFLGELEEILDVIEPSQFVKIQEPLFKQIAKCVSSPHFQVAERALYYWNNEYIMS    374
gi|586990135:195-1598           SQKEVMFLGELEEILDVIEPSQFVKIQEPLFKQIAKCVSSPHFQVAERALYYWNNEYIMS    374
gi|532164714:591-1994           SQKEVMFLGELEEILDVIEPSQFVKIQEPLFKQIAKCVSSPHFQVAERALYYWNNEYIMS    374
gi|134085748:529-1932           SQKEVMFLGELEEILDVIEPSQFVKIQEPLFKQIAKCVSSPHFQVAERALYYWNNEYIMS    374
gi|426233507:51-1454            SQKEVMFLGELEEILDVIEPSQFVKIQEPLFKQIAKCVSSPHFQVAERALYYWNNEYIMS    374
gi|55741699:574-1977            SQKEVMFLGELEEILDVIEPSQFVKIQEPLFKQIAKCVSSPHFQVAERALYYWNNEYIMS    374
gi|672056393:586-1989           SQKEVMFLGELEEILDVIEPSQFVKIQEPLFKQIAKCVSSPHFQVAERALYYWNNEYIMS    374
gi|528511094:406-1860           SQKEVMFLGEIEEILDVIEPTQFKKIQEPLFKQIAKCVASPHFQVAERALYYWNNEYILS    374
gi|657570088:928-2469           SQKEVMFLGEIEEILDVIEPTQFKKIQEPLFKQISKCVANPHFQVAERALYFWNNEYILS    371
gi|557292702:1-1446             SQKEVMFLGEIEEILDVIEPTQFKKIEEPLFKQISKCVSSSHFQVAERALYFWNNEYILS    376
gi|641796061:534-1967           SQKEVMFLGEIEEILDVIEPTQFKKIEEPLFKQISKCVSSSHFQVAERALYFWNNEYILS    372
gi|118130061:626-2086           SQKEVMFLGEIEEILDVIEPTQFKKIEEPLFKQISKCVSSSHFQVAERALYFWNNEYILS    382
gi|157823929:178-1638           SQKEVMFLGEIEEILDVIEPTQFKKIEEPLFKQISKCVSSSHFQVAERALYFWNNEYILS    382
```

```
gi|126165211:499-1968            SQKEVMFLGEIEEILDVIEPTQFKKIEEPLFKQISKCVSSSHFQVAERALYFWNNEYILS      385
gi|426240097:195-1664            SQKEVMFLGEIEEILDVIEPTQFKKIEEPLFKQISKCVSSSHFQVAERALYFWNNEYILS      385
gb|L42373.1|HUMPP2A:572-2032     SQKEVMFLGEIEEILDVIEPTQFKKIEEPLFKQISKCVSSSHFQVAERALYFWNNEYILS      382
gi|755806859:84-1547             SQKEVMFLGEIEEILDVIEPTQFKKIEEPLFKQISKCVSSSHFQVAERALYFWNNEYILS      383
                                 : ****** *:*********::* :: : **:*:: *::. **********:******:*

gi|530584065:87-1661             LISDNAAKILPIMFPSLYRNSKTHWNKTIHGLIYNALKLFMEMNQKLFDDCTQQFKAEKL      417
gi|557265256:74-1648             LISDNAAKILPIMFPSLYRNSKTHWNKTIHGLIYNALKLFMEMNQKLFDDCTQQFKAEKL      417
gi|755745075:318-1892            LISDNAAKILPIMFPSLYRNSKTHWNKTIHGLIYNALKLFMEMNQKLFDDCTQQFKAEKL      417
gi|555290051:121-1695            LISDNAAKILPIMFPSLYRNSKTHWNKTIHGLIYNALKLFMEMNQKLFDDCTQQFKAEKL      417
gi|300798097:82-1656             LISDNAAKILPIMFPSLYRNSKTHWNKTIHGLIYNALKLFMEMNQKLFDDCTQQFKAEKL      417
gi|240849335:141-1715            LISDNAAKILPIMFPSLYRNSKTHWNKTIHGLIYNALKLFMEMNQKLFDDCTQQFKAEKL      417
gi|803180317:2708-4297           LISDNAAKILPIMFPSLYRNSKTHWNKTIHGLIYNALKLFMEMNQKLFDDCTQQFKAEKL      417
gi|134085945:20-1594             LISDNAAKILPIMFPSLYRNSKTHWNKTIHGLIYNALKLFMEMNQKLFDDCTQQFKAEKL      417
gi|688603875:111-1688            LISDNAAKILPIMFPSLYRNSKTHWNKTIHGLIYNALKLFMEMNQKLFDDCTQQFRAEKN      417
gi|657585651:111-1688            LISDNAAKILPIMFPALYRNSKTHWNKTIHGLIYNALKLFMEMNQKLFDDCTQQFRAEKS      417
gi|657585641:75-1811             LISDNAAKILPIMFPALYRNSKTHWNKTIHGLIYNALKLFMEMNQKLFDDCTQQFRAEKS      470
gi|50726891:63-1868              LISDNAAKILPIMFPALYKNSKSHWNKTIHGLIYNALKLFMEMNQKLFDDCTQQYKAEKQ      480
gi|426251100:1-1800              LISDNAARVLPIMFPALYRNSKSHWNKTIHGLIYNALKLFMEMNQKLFDDCTQQYKAEKQ      490
gi|14669809:89-1873              LISDNAARILPIMFPALYRNSKSHWNKTIHGLIYNALKLFMEMNQKLFDDCTQQYKAEKQ      485
gi|672023745:791-2578            LISDNAARVLPIMFPALYRNSKSHWNKTIHGLIYNALKLFMEMNQKLFDDCTQQYKAEKQ      486
gb|L76702.1|HUMB56DA:188-1996    LISDNAARVLPIMFPALYRNSKSHWNKTIHGLIYNALKLFMEMNQKLFDDCTQQYKAEKQ      493
gi|329744635:107-1909            LISDNAARVLPIMFPALYRNSKSHWNKTIHGLIYNALKLFMEMNQKLFDDCTQQYKAEKQ      491
gi|755728309:140-1948            LISDNAARVLPIMFPALYRNSKSHWNKTIHGLIYNALKLFMEMNQKLFDDCTQQYKAEKQ      493
gi|641758569:96-1892             LISDNAAKILPIMFPALYKNSKSHWNKTIHGLIYNALKLFMEMNQKLFDDCTQQYKAEKQ      489
gi|557295947:96-1958             LISDNAAKILPIMFPALYKNSKSHWNKTIHGLIYNALKLFMEMNQKLFDDCTQQYKAEKQ      511
gi|688564053:1400-2911           LIEENCQVILPLVFGTLYRVSKEHWNQTIISLIYNVLKTFMEMNSKLFDDLTASYKVEKQ      447
gi|657563479:1-1563              LIEENCQVILPLVFATLYRVSKEHWNQTIVSLIYNVLKTFMEMNSKLFDDLTASYKVEKQ      446
gi|115497699:359-1849            LIEDNCHTVLPAVFGTLYQVSKEHWNQTIVSLIYNVLKTFMEMNGKLFDELTASYKLEKQ      448
gi|803191297:577-2067            LIEDNCHTVLPAVFGTLYQVSKEHWNQTIVSLIYNVLKTFMEMNGKLFDELTASYKLEKQ      448
gi|60593006:48-1541              LIEDNCHTVLPAVFGTLYQVSKEHWNQTIVSLIYNVLKTFMEMNGKLFDELTASYKLEKQ      448
gi|142364854:598-2091            LIEDNCHTVLPAVFGTLYQVSKEHWNQTIVSLIYNVLKTFMEMNGKLFDELTASYKLEKQ      448
gb|L42374.1|HUMPP2ABA:326-1819   LIEDNCHTVLPAVFGTLYQVSKEHWNQTIVSLIYNVLKTFMEMNGKLFDELTASYKLEKQ      448
gi|587003404:628-2121            LIEDNCHTVLPAVFGTLYQVSKEHWNQTIVSLIYNVLKTFMEMNGKLFDELTASYKLEKQ      448
gi|641793336:1879-3372           LIEDNCHTVLPAIFGTLYRVSKEHWNQTIVSLVYNVLKTFMEMNGKLFDELTASYKVEKQ      448
gi|557320144:379-1860            LIEDNCQAVLPAIFGTLYRVSKEHWNQTIVSLVYNVLKTFMEMNGKLFDELTASYKLEKQ      448
gi|51491868:454-1860             LIEENCSAILPIMFASLYRISKEHWNPAISALIYNVLKAFMEMNSALFDELAASYKSDRQ      434
gi|657554348:108-1517            LIEENSSVILPIMFASLYRISKEHWNPAIVALVYNVLKAFMEMNSTLFDELTATYKSDRQ      436
gi|641761822:526-1914            LIEENSNVILPIMFSSLYRISKEHWNPAIVALVYNVLKAFMEMNSTMFDELTATYN----      430
gi|557266651:182-1570            LIEENSNVILPIMFSSLYRISKEHWNPAIVALVYNVLKAFMEMNSTMFDELTATYN----      430
gi|586990135:195-1598            LIEENSNVILPIMFSSLYRIXKEHWNPAIVALVYNVLKAFMEMNSTMFDELTATYKSDRQ      434
gi|532164714:591-1994            LIEENSNVILPIMFSSLYRISKEHWNPAIVALVYNVLKAFMEMNSTMFDELTATYKSDRQ      434
gi|134085748:529-1932            LIEENSNVILPIMFSSLYRISKEHWNPAIVALVYNVLKAFMEMNSTMFDELTATYKSDRQ      434
gi|426233507:51-1454             LIEENSNVILPIMFSSLYRISKEHWNPAIVALVYNVLKAFMEMNSTMFDELTATYKSDRQ      434
gi|55741699:574-1977             LIEENSNVILPIMFSSLYRISKEHWNPAIVALVYNVLKAFMEMNSTMFDELTATYKSDRQ      434
gi|672056393:586-1989            LIEENSNVILPIMFSSLYRISKEHWNPAIVALVYNVLKAFMEMNSTMFDELTATYKSDRQ      434
```

```
gi|528511094:406-1860               LIEENNAKIFPIMFGNLYRISKEHWNPTIVALVYNVLKTMMEMNSKLFDELTTSYKSDRQ	434
gi|657570088:928-2469               LIEENIDKVLPIMFGSLYRISKEHWNPTIVALVYNVLKTLMEMNCTLFDELTSSYKADRQ	431
gi|557292702:1-1446                 LIEENIDKILPIMFGSLYKISKEHWNPTIVALVYNVLKTLMEMNGKLFDELTSTYKAERQ	436
gi|641796061:534-1967               LIEENINKILPIMFGSLYKISKEHWNPTIVALVYNVLKTLMEMNGKLFDELTSSYKAERQ	432
gi|118130061:626-2086               LIEENIDKILPIMFASLYKISKEHWNQTIVALVYNVLKTLMEMNGKLFDDLTSSYKAERQ	442
gi|157823929:178-1638               LIEENIDKILPIMFASLYKISKEHWNQTIVALVYNVLKTLMEMNGKLFDDLTSSYKAERQ	442
gi|126165211:499-1968               LIEENIDKILPIMFGSLYKISKEHWNPTIVALVYNVLKTLMEMNGKLFDDLTSSYKAERQ	445
gi|426240097:195-1664               LIEENIDKILPIMFGSLYKISKEHWNPTIVALVYNVLKTLMEMNGKLFDDLTSSYKAERQ	445
gb|L42373.1|HUMPP2A:572-2032        LIEENIDKILPIMFASLYKISKEHWNPTIVALVYNVLKTLMEMNGKLFDDLTSSYKAERQ	442
gi|755806859:84-1547                LIEENIDKILPIMFGSLYKISKEHWNPTIVALVYNVLKTLMEMNGKLFDDLTSSYKAERQ	443
                                    **.:*   ::* :*  **:  * *** :* .*:**.** :****  :**: :  :.

gi|530584065:87-1661                KEKLKSKEREEAWVKIENLAKSNPQYPTYSDTSV------------LNSPVAMETDGPLI	465
gi|557265256:74-1648                KEKLKLKEREEAWVKIENLAKSNPQYPVYSDTSV------------LNSPVAMETDGPLI	465
gi|755745075:318-1892               KEKLKMKEREEAWVKIENLAKANPQHAVCGEANT------------VSLPVAMGTDGPLS	465
gi|555290051:121-1695               KEKLKMKEREEAWVKIENLAKANPQYAVYSQASA------------VSIPVAMETDGPQF	465
gi|300798097:82-1656                KEKLKMKEREEAWVKIENLAKANPQYAVYSQASA------------MSIPVAMETDGPQF	465
gi|240849335:141-1715               KEKLKMKEREEAWVKIENLAKANPQYTVYSQAST------------MSIPVAMETDGPLF	465
gi|803180317:2708-4297              KEKLKMKEREEAWVKIENLAKANPQYAVYSQAST------------ISIPVAMETDGPLF	465
gi|134085945:20-1594                KEKLKMKEREEAWVKIENLAKANPQYAVYSQAST------------ISIPVAMETDGPLF	465
gi|688603875:111-1688               KEKAKWKEREEAWIKIENLAKSNPQFLMYIDANS------------LCSPMDMETDGPML	465
gi|657585651:111-1688               KEKAKWKEREEAWLKIENLAKSNPQFLTYVDSIG------------SGSPMDMETDGPLL	465
gi|657585641:75-1811                KEKAKWKEREEAWLKIENLAKSNPQFLTYVDSIG------------SGSPMDMETDGPLL	518
gi|50726891:63-1868                 KEKYKVKEREEMWHKIEALAKQNPQSTKVQLRPGLAQEEYMMYNEGGMPIYSMETETPTV	540
gi|426251100:1-1800                 KGRFRMKEREEMWQKIEELARLNPQYPMFRAPPP------------LPPVYSMETETPTA	538
gi|14669809:89-1873                 KGRFRMKEREEMWQKIEELARLNPQYPMFRAPPP------------LPPVYSMETETPTA	533
gi|672023745:791-2578               KGRFRMKEREEMWQKIEELARLNPQYPMFRAPPP------------LPPVYSMETETPTA	534
gb|L76702.1|HUMB56DA:188-1996       KGRFRMKEREEMWQKIEELARLNPQYPMFRAPPP------------LPPVYSMETETPTA	541
gi|329744635:107-1909               KGRFRMKEREEMWQKIEELARLNPQYPMFRAPPP------------LPPVYSMETETPTA	539
gi|755728309:140-1948               KGRFRMKEREEMWQKIEELARLNPQYPMFRAPPP------------LPPVYSMETETPTA	541
gi|641758569:96-1892                KGRFKLREREEMWHKVEELARQNPQYPMYYAPPP------------LPPVYCMETETPTA	537
gi|557295947:96-1958                KGRFRMKERVEMWQKIEELARLNPQYPMYYAPPP------------LPPVYCMETETPTA	559
gi|688564053:1400-2911              KELKKERERVELWRNLEDLRERRLQSLTEASRNQKNLQERENTAKSQSDT----SAANTT	503
gi|657563479:1-1563                 KELKRERERAELWRGLEEHQERRMQMLTEAARNQRNLQERGERGDPLTPS----SPQTAH	502
gi|115497699:359-1849               QEQQKARERQELWQGLEELRLRRLQGTQGAKEAPLQRLTPQVTGGGQS*-----------	496
gi|803191297:577-2067               QEQQKARERQELWQGLEELRLRRLQGTQGAKEAPLQRLTPQVTGGGQS*-----------	496
gi|60593006:48-1541                 QEQQKAQERQELWRGLEELRLRRLQGTQGAKEAPVPRPTPQVAASGGQS*----------	497
gi|142364854:598-2091               QEQQKAQERQELWRGLEELRLRRLQGTQGAKEAPVPRPTPQVAASGGQS*----------	497
gb|L42374.1|HUMPP2ABA:326-1819      QEQQKAQERQELWQGLEELRLRRLQGTQGAKEAPLQRLTPQVAASGGQS*----------	497
gi|587003404:628-2121               QEQQKARERQELWQGLEELRLRRLQGTQGAKEAPLQRLTPQVATSGGQS*----------	497
gi|641793336:1879-3372              QELKKEKERQELWKQLDELQLKKLQGLEEAQMNRLNLQHSLGLQSGNKS*----------	497
gi|557320144:379-1860               QELKKEKERQELWRQLDELRLRRLRGLEEAQMNRLNLQHGHSPKT*--------------	493
gi|51491868:454-1860                RERKKDKEREDLWRKLEDLELRRGIQNSDGVIPT*-------------------------	468
gi|657554348:108-1517               REKKKEKEREELWKKLEDLELKRGLRSDGIIPT*--------------------------	469
gi|641761822:526-1914               -EKKKEKEREELWKKLEDLELKRGLRRDGIIPT*--------------------------	462
gi|557266651:182-1570               -EKKKEKEREELWKKLEDLELKRGLRRDGIIPT*--------------------------	462
```

```
gi|586990135:195-1598               REKKKEKEREELWKKLEDLELKRGLRRDGIIPT*--------------------------   467
gi|532164714:591-1994               REKKKEKEREELWKKLEDLELKRGLRRDGIIPT*--------------------------   467
gi|134085748:529-1932               REKKKEKEREELWKKLEDLELKRGLRRDGIIPT*--------------------------   467
gi|426233507:51-1454                REKKKEKEREELWKKLEDLELKRGLRRDGIIPT*--------------------------   467
gi|55741699:574-1977                REKKKEKEREELWKKLEDLELKRGLRRDGIIPT*--------------------------   467
gi|672056393:586-1989               REKKKEKEREELWKKLEDLELKRGLRRDGIIPT*--------------------------   467
gi|528511094:406-1860               REKKKEQEREELWRRLDELRMKEVLMIQNNRH-DIHSLTTTTSIKNNEH-----DI*---   484
gi|657570088:928-2469               REKKKEQERDELWKKLEELRLSNATLVQNNSH-GLPSVQNNNNNNNNS-----NNDSVN   485
gi|557292702:1-1446                 REKKKELEREELWRKLEELKLKKVLAEKQNSTHNVLNAQNNTSAK*--------------   481
gi|641796061:534-1967               REKKKELEREELWRKLEELKLKKTLVEKQNSTHNVLNAHNSTSAK*--------------   477
gi|118130061:626-2086               REKKKELEREELWKKLEELQLKKALEKQNNA-YNMHSIRSSTSAK*--------------   486
gi|157823929:178-1638               REKKKELEREELWKKLEELKLKKALEKQNNA-YNMHSILSNTSAQ*--------------   486
gi|126165211:499-1968               REKKKELEREELWKKLEELKLKKALEKQNSA-YNMHSILSNTNDG*--------------   489
gi|426240097:195-1664               REKKKELEREELWKKLEELKLKKALEKQNSA-YNMHSILSNTNDG*--------------   489
gb|L42373.1|HUMPP2A:572-2032        REKKKELEREELWKKLEELKLKKALEKQNSA-YNMHSILSNTSAE*--------------   486
gi|755806859:84-1547                REKKKELEREELWKKLEELKLKKALEKQNSA-YNMHSILSNTSDE*--------------   487
                                        :  ** : *  ::     .

gi|530584065:87-1661                EDLQMLKKTVKEEACQAQRDQKKDRPLMRRKSELPQDIYTMKALESHCRADELISH-DGH   524
gi|557265256:74-1648                EDLQMLKKTVKEEACQAQKDQKKDRPLVRRKSELPQDIYTMKALESHCRADELISH-DGH   524
gi|755745075:318-1892               EDVQTLRETVSGQARQAQKDLKKDRPLVRRKSELPQDLHTKNALEAHCRADQLVSQ-DGR   524
gi|555290051:121-1695               EDVQMLKKTVSDEARQAQKELKKDRPLVRRKSELPQDPHTEKALEAHCRASELLSQ-DGR   524
gi|300798097:82-1656                EDVQMLKKTVSDETRQAQKDLKKDRPLVRRKSELPQDPHTEKALEAHCRASELLSQ-DGR   524
gi|240849335:141-1715               EDVQMLRKTVKDEAHQAQKDPKKDRPLARRKSELPQDPHTKKALEAHCRADELASQ-DGR   524
gi|803180317:2708-4297              EDVQMLRKTVNEEARQAQKDPKKERPLVRRKSDLPQDLHAKSALEAHGRAEEPAPQ-DGR   524
gi|134085945:20-1594                EDVQMLRKTVNEEARQAQKDPKKERPLARRKSELPQDLHTKSALEAHGRAEEPAPQ-DGR   524
gi|688603875:111-1688               EDVLMLKKTVEEEATPLHREQRKERPLMRRKSELPQDTSTVKALETHRRAEDMIGTQDGH   525
gi|657585651:111-1688               DDVNLLKKTVAEEATQIQKDQRRERPLMRRKSELPKDISTVTALELHRRAEEMLTTHDGH   525
gi|657585641:75-1811                DDVNLLKKTVAEEATQIQKDQRRERPLMRRKSELPKDISTVTALELHRRAEEMLTTHDGH   578
gi|50726891:63-1868                 EDIQLLKKTVESEASQGMKEIKKDKVLMRRKSELPQDVYTIKALEAHKRAEEYLTANQEA   600
gi|426251100:1-1800                 EDIQLLKRTVETEAVQMLKDIKKEKVLLRRKSELPQDVYTIKALEAHKRAEEFLTASQEA   598
gi|14669809:89-1873                 EDIQLLKRTVETEAVQMLKDIKKDKVLLRRKSELPQDVYTIKALEAHKRAEEFLTASQEA   593
gi|672023745:791-2578               EDIQLLKRTVETEAVQMLKDIKKDKVLLRRKSELPQDVYTIKALEAHKRAEEFLTASQEA   594
gb|L76702.1|HUMB56DA:188-1996       EDIQLLKRTVETEAVQMLKDIKKEKVLLRRKSELPQDVYTIKALEAHKRAEEFLTASQEA   601
gi|329744635:107-1909               EDIQLLKRTVETEAVQMLKDIKKEKVLLRRKSELPQDVYTIKALEAHKRAEEFLTASQEA   599
gi|755728309:140-1948               EDIQLLKRTVETEAVQMLKDIKKEKVLLRRKSELPQDVYTIKALEAHKRAEEFLTASQEA   601
gi|641758569:96-1892                EDIQLLKKTVETEAVQMLKDIKKEKVLLRRKSELPQDVYTIKALEAHKRAEEFLTSSQEA   597
gi|557295947:96-1958                EDIQLLKKTMETEAVQMLKDIKKEKVLLHRKSELPQDVYTIKALEAHKRAEEFLTSSQEA   619
gi|688564053:1400-2911              *-----------------------------------------------------------   503
gi|657563479:1-1563                 SDQPEPKPSGASSGES-------AT*----------------------------------   520
gi|115497699:359-1849               ------------------------------------------------------------   496
gi|803191297:577-2067               ------------------------------------------------------------   496
gi|60593006:48-1541                 ------------------------------------------------------------   497
gi|142364854:598-2091               ------------------------------------------------------------   497
gb|L42374.1|HUMPP2ABA:326-1819      ------------------------------------------------------------   497
gi|587003404:628-2121               ------------------------------------------------------------   497
```

```
gi|641793336:1879-3372          ------------------------------------------------------------      497
gi|557320144:379-1860           ------------------------------------------------------------      493
gi|51491868:454-1860            ------------------------------------------------------------      468
gi|657554348:108-1517           ------------------------------------------------------------      469
gi|641761822:526-1914           ------------------------------------------------------------      462
gi|557266651:182-1570           ------------------------------------------------------------      462
gi|586990135:195-1598           ------------------------------------------------------------      467
gi|532164714:591-1994           ------------------------------------------------------------      467
gi|134085748:529-1932           ------------------------------------------------------------      467
gi|426233507:51-1454            ------------------------------------------------------------      467
gi|55741699:574-1977            ------------------------------------------------------------      467
gi|672056393:586-1989           ------------------------------------------------------------      467
gi|528511094:406-1860           ------------------------------------------------------------      484
gi|657570088:928-2469           ED---KSADGAAETVD-------GKDTME-------------A---------------SE      507
gi|557292702:1-1446             ------------------------------------------------------------      481
gi|641796061:534-1967           ------------------------------------------------------------      477
gi|118130061:626-2086           ------------------------------------------------------------      486
gi|157823929:178-1638           ------------------------------------------------------------      486
gi|126165211:499-1968           ------------------------------------------------------------      489
gi|426240097:195-1664           ------------------------------------------------------------      489
gb|L42373.1|HUMPP2A:572-2032    ------------------------------------------------------------      486
gi|755806859:84-1547            ------------------------------------------------------------      487


gi|530584065:87-1661            *------     524
gi|557265256:74-1648            *------     524
gi|755745075:318-1892           *------     524
gi|555290051:121-1695           *------     524
gi|300798097:82-1656            *------     524
gi|240849335:141-1715           *------     524
gi|803180317:2708-4297          PRACP*-     529
gi|134085945:20-1594            *------     524
gi|688603875:111-1688           *------     525
gi|657585651:111-1688           *------     525
gi|657585641:75-1811            *------     578
gi|50726891:63-1868             L*-----     601
gi|426251100:1-1800             L*-----     599
gi|14669809:89-1873             L*-----     594
gi|672023745:791-2578           L*-----     595
gb|L76702.1|HUMB56DA:188-1996   L*-----     602
gi|329744635:107-1909           L*-----     600
gi|755728309:140-1948           L*-----     602
gi|641758569:96-1892            L*-----     598
gi|557295947:96-1958            L*-----     620
gi|688564053:1400-2911          -------     503
gi|657563479:1-1563             -------     520
```

```
gi|115497699:359-1849             -------     496
gi|803191297:577-2067             -------     496
gi|60593006:48-1541               -------     497
gi|142364854:598-2091             -------     497
gb|L42374.1|HUMPP2ABA:326-1819    -------     497
gi|587003404:628-2121             -------     497
gi|641793336:1879-3372            -------     497
gi|557320144:379-1860             -------     493
gi|51491868:454-1860              -------     468
gi|657554348:108-1517             -------     469
gi|641761822:526-1914             -------     462
gi|557266651:182-1570             -------     462
gi|586990135:195-1598             -------     467
gi|532164714:591-1994             -------     467
gi|134085748:529-1932             -------     467
gi|426233507:51-1454              -------     467
gi|55741699:574-1977              -------     467
gi|672056393:586-1989             -------     467
gi|528511094:406-1860             -------     484
gi|657570088:928-2469             STPCAK*     513
gi|557292702:1-1446               -------     481
gi|641796061:534-1967             -------     477
gi|118130061:626-2086             -------     486
gi|157823929:178-1638             -------     486
gi|126165211:499-1968             -------     489
gi|426240097:195-1664             -------     489
gb|L42373.1|HUMPP2A:572-2032      -------     486
gi|755806859:84-1547              -------     487
```
