# Supplementary material for: A Nonsynonymous/Synonymous Substitution Analysis of the B56 Gene Family Aids in Understanding B56 Isoform Diversity
Source: PLoS One. 2015 Dec 21;10(12):e0145529. doi: 10.1371/journal.pone.0145529 (PMC4687035; doi:10.1371/journal.pone.0145529)
Supplement: S1 Table — The means and standard deviations from dN/dS analyses for the family-wide, B56-1, B56-2, and individual isoform groupings are provided. (DOCX) [file pone.0145529.s008.docx]

|  | dN | | dS | | dN/dS | |
| --- | --- | --- | --- | --- | --- | --- |
|  | mean | std | mean | std | mean | std |
| all  B56-1(αβε)  B56-2(γδ)  α  β  γ  δ  δ/γ  ε | 0.2264  0.1549  0.1188  0.0617  0.0770  0.0440  0.0698  0.0646  0.0174 | 0.1034  0.0770  0.0704  0.0447  0.0557  0.0263  0.0602  0.0432  0.0228 | 1.5654  1.1368  1.5020  0.7546  0.8634  0.6897  1.5918  0.5643  0.3477 | 1.1324  0.9642  1.1358  0.8919  0.4463  0.3347  1.0584  0.3418  0.2592 | 0.1184  0.0656  0.0545  0.0504  0.0739  0.0391  0.0418  0.0552  0.0046 | 0.1243  0.0489  0.0816  0.0300  0.0405  0.0096  0.0283  0.0112  0.0056 |
