# Supplementary material for: A Nonsynonymous/Synonymous Substitution Analysis of the B56 Gene Family Aids in Understanding B56 Isoform Diversity
Source: PLoS One. 2015 Dec 21;10(12):e0145529. doi: 10.1371/journal.pone.0145529 (PMC4687035; doi:10.1371/journal.pone.0145529)
Supplement: S2 Table — p values from dN/dS analyses for the family-wide, B56-1, B56-2, and individual isoform groupings are provided. p values less than 0.05 are highlighted in yellow. (DOCX) [file pone.0145529.s009.docx]

| **QUERY** | **SUBJECT** | **dN** | **dS** | **dN/dS** |
| --- | --- | --- | --- | --- |
| ALL | B56-1 | 0.0000 | 0.0000 | 0.0000 |
| ALL | B56-2 | 0.0000 | 0.2710 | 0.0000 |
| ALL | α | 0.0000 | 0.0000 | 0.0002 |
| ALL | β | 0.0001 | 0.0000 | 0.0299 |
| ALL | γ | 0.0000 | 0.0000 | 0.0000 |
| ALL | δ | 0.0000 | 0.9415 | 0.0000 |
| ALL | δ/γ | 0.0000 | 0.0001 | 0.0325 |
| ALL | ε | 0.0000 | 0.0000 | 0.0000 |
| B56-1 | B56-2 | 0.6938 | 0.0099 | 0.0322 |
| B56-1 | α | 0.0452 | 0.0287 | 0.2005 |
| B56-1 | β | 0.7159 | 0.3437 | 0.1868 |
| B56-1 | γ | 0.0450 | 0.0629 | 0.0421 |
| B56-1 | δ | 0.5188 | 0.0047 | 0.0172 |
| B56-1 | δ/γ | 0.1580 | 0.0336 | 0.5803 |
| B56-1 | ε | 0.0000 | 0.0000 | 0.0000 |
| B56-2 | α | 0.0094 | 0.0010 | 0.6336 |
| B56-2 | β | 0.9299 | 0.4387 | 0.0016 |
| B56-2 | γ | 0.0027 | 0.0026 | 0.4101 |
| B56-2 | δ | 0.7444 | 0.7219 | 0.1051 |
| B56-2 | δ/γ | 0.0404 | 0.0015 | 0.0074 |
| B56-2 | ε | 0.0000 | 0.0000 | 0.0000 |
| α | β | 0.0054 | 0.0335 | 0.0195 |
| α | γ | 0.8887 | 0.5037 | 0.2763 |
| α | δ | 0.0291 | 0.0013 | 0.1269 |
| α | δ/γ | 0.7669 | 0.5298 | 0.1095 |
| α | ε | 0.0000 | 0.0011 | 0.0000 |
| β | γ | 0.0047 | 0.0802 | 0.0069 |
| β | δ | 0.4320 | 0.0006 | 0.0005 |
| β | δ/γ | 0.0242 | 0.0274 | 0.1063 |
| β | ε | 0.0000 | 0.0000 | 0.0000 |
| γ | δ | 0.0065 | 0.0025 | 0.3646 |
| γ | δ/γ | 0.6102 | 0.1963 | 0.0000 |
| γ | ε | 0.0000 | 0.0000 | 0.0000 |
| δ | δ/γ | 0.0427 | 0.0020 | 0.0014 |
| δ | ε | 0.0000 | 0.0000 | 0.0000 |
| δ/γ | ε | 0.0000 | 0.0057 | 0.0000 |
