# Supplementary material for: A Nonsynonymous/Synonymous Substitution Analysis of the B56 Gene Family Aids in Understanding B56 Isoform Diversity
Source: PLoS One. 2015 Dec 21;10(12):e0145529. doi: 10.1371/journal.pone.0145529 (PMC4687035; doi:10.1371/journal.pone.0145529)
Supplement: S3 Table — The span of the N-terminus, core, and C-terminus for the family-wide, B56-1, B56-2, and individual isoform groupings from alignments generated in dN/dS analyses are provided. (DOCX) [file pone.0145529.s010.docx]

|  | N-terminus | Core | C-terminus |
| --- | --- | --- | --- |
| all  B56-1(αβε)  B56-2(γδ)  α  β  γ  δ  δ/γ  ε | 1-142  1-74  1-138  1-67  1-70  1-32  1-138  1-90  1-55 | 143-529  75-461  139-526  68-449  71-452  33-418  139-525  91-476  56-437 | 530-679  462-544  527-650  450-532  453-526  419-531  526-646  477-609  438-471 |
