# Supplementary material for: A Nonsynonymous/Synonymous Substitution Analysis of the B56 Gene Family Aids in Understanding B56 Isoform Diversity
Source: PLoS One. 2015 Dec 21;10(12):e0145529. doi: 10.1371/journal.pone.0145529 (PMC4687035; doi:10.1371/journal.pone.0145529)
Supplement: S4 Table — The means and standard deviations from dN/dS analyses for the family-wide, B56-1, B56-2, and individual isoform groupings are provided. (DOCX) [file pone.0145529.s011.docx]

|  | dN | | dS | | dN/dS | |
| --- | --- | --- | --- | --- | --- | --- |
|  | mean | std | mean | std | mean | std |
| all  B56-1(αβε)  B56-2(γδ)  α  β  γ  δ  δ/γ  ε | 0.1498  0.1149  0.0430  0.0308  0.0457  0.0079  0.0304  0.0087  0.0154 | 0.0832  0.0609  0.0301  0.0316  0.0365  0.0065  0.0235  0.0071  0.0201 | 1.4997  1.1036  1.3242  0.6253  0.8928  0.7703  1.4722  0.6306  0.3702 | 1.0737  0.9556  0.8751  0.4394  0.4997  0.3513  0.9340  0.3553  0.2919 | 0.0579  0.0362  0.0164  0.0110  0.0414  0.0042  0.0204  0.0039  0.0031 | 0.0589  0.0346  0.0164  0.0082  0.0269  0.0019  0.0177  0.0020  0.0034 |
