# Supplementary material for: A Nonsynonymous/Synonymous Substitution Analysis of the B56 Gene Family Aids in Understanding B56 Isoform Diversity
Source: PLoS One. 2015 Dec 21;10(12):e0145529. doi: 10.1371/journal.pone.0145529 (PMC4687035; doi:10.1371/journal.pone.0145529)
Supplement: S5 Table — p values from dN/dS analyses for the family-wide, B56-1, B56-2, and individual isoform groupings are provided. p values less than 0.05 are highlighted in yellow. (DOCX) [file pone.0145529.s012.docx]

| **QUERY** | **SUBJECT** | **dN** | **dS** | **dN/dS** |
| --- | --- | --- | --- | --- |
| ALL | B56-1 | 0.0000 | 0.0000 | 0.0000 |
| ALL | B56-2 | 0.0000 | 0.1601 | 0.0000 |
| ALL | α | 0.0000 | 0.0000 | 0.0000 |
| ALL | β | 0.0009 | 0.0000 | 0.0670 |
| ALL | γ | 0.0000 | 0.0000 | 0.0000 |
| ALL | δ | 0.0001 | 0.9170 | 0.0001 |
| ALL | δ/γ | 0.0000 | 0.0001 | 0.0000 |
| ALL | ε | 0.0000 | 0.0000 | 0.0000 |
| B56-1 | B56-2 | 0.1582 | 0.0089 | 0.0017 |
| B56-1 | α | 0.0300 | 0.0339 | 0.0322 |
| B56-1 | β | 0.6803 | 0.7267 | 0.1757 |
| B56-1 | γ | 0.0022 | 0.4271 | 0.0000 |
| B56-1 | δ | 0.6589 | 0.0097 | 0.3125 |
| B56-1 | δ/γ | 0.0045 | 0.1610 | 0.0001 |
| B56-1 | ε | 0.0000 | 0.0000 | 0.0000 |
| B56-2 | α | 0.0357 | 0.0002 | 0.4462 |
| B56-2 | β | 0.0110 | 0.0045 | 0.0000 |
| B56-2 | γ | 0.0002 | 0.0089 | 0.0000 |
| B56-2 | δ | 0.1779 | 0.5650 | 0.1267 |
| B56-2 | δ/γ | 0.0008 | 0.0028 | 0.0009 |
| B56-2 | ε | 0.0000 | 0.0000 | 0.0000 |
| α | β | 0.0002 | 0.0210 | 0.0001 |
| α | γ | 0.0065 | 0.0751 | 0.0000 |
| α | δ | 0.0001 | 0.0012 | 0.0013 |
| α | δ/γ | 0.0081 | 0.7419 | 0.0000 |
| α | ε | 0.0001 | 0.0044 | 0.0000 |
| β | γ | 0.0000 | 0.3639 | 0.0000 |
| β | δ | 0.0501 | 0.0016 | 0.0017 |
| β | δ/γ | 0.0001 | 0.1063 | 0.0000 |
| β | ε | 0.0000 | 0.0000 | 0.0000 |
| γ | δ | 0.0000 | 0.0076 | 0.0000 |
| γ | δ/γ | 0.5767 | 0.1202 | 0.9716 |
| γ | ε | 0.0001 | 0.0000 | 0.0077 |
| δ | δ/γ | 0.0000 | 0.0060 | 0.0000 |
| δ | ε | 0.0000 | 0.0000 | 0.0000 |
| δ/γ | ε | 0.0228 | 0.0046 | 0.0439 |
