# Supplementary material for: A Nonsynonymous/Synonymous Substitution Analysis of the B56 Gene Family Aids in Understanding B56 Isoform Diversity
Source: PLoS One. 2015 Dec 21;10(12):e0145529. doi: 10.1371/journal.pone.0145529 (PMC4687035; doi:10.1371/journal.pone.0145529)
Supplement: S6 Table — The means and standard deviations from dN/dS analyses for the family-wide, B56-1, B56-2, and individual isoform groupings are provided. (DOCX) [file pone.0145529.s013.docx]

|  | dN | | dS | | dN/dS | |
| --- | --- | --- | --- | --- | --- | --- |
|  | mean | std | mean | std | mean | std |
| all  B56-1(αβε)  B56-2(γδ)  α  β  γ  δ  δ/γ  ε | 0.9716  0.3108  0.9220  0.1990  0.0898  0.0897  0.1726  0.2617  0.0225 | 0.6238  0.1741  0.7725  0.1609  0.0868  0.0840  0.1250  0.1877  0.0260 | 1.5137  1.1570  1.4279  0.7448  0.8255  0.6087  1.4053  0.7592  0.6362 | 0.8432  0.7303  0.9814  0.5063  0.4681  0.4689  1.0370  0.6824  0.7747 | 0.7283  0.2517  0.4943  0.3183  0.0861  0.1720  0.1329  0.2504  0.0243 | 0.6300  0.1779  0.4901  0.2114  0.0816  0.1325  0.0757  0.1014  0.0202 |
