# Supplementary material for: A Nonsynonymous/Synonymous Substitution Analysis of the B56 Gene Family Aids in Understanding B56 Isoform Diversity
Source: PLoS One. 2015 Dec 21;10(12):e0145529. doi: 10.1371/journal.pone.0145529 (PMC4687035; doi:10.1371/journal.pone.0145529)
Supplement: S7 Table — p values from dN/dS analyses for the family-wide, B56-1, B56-2, and individual isoform groupings are provided. p values less than 0.05 are highlighted in yellow. (DOCX) [file pone.0145529.s014.docx]

| **QUERY** | **SUBJECT** | **dN** | **dS** | **dN/dS** |
| --- | --- | --- | --- | --- |
| ALL | B56-1 | 0.0000 | 0.0000 | 0.0000 |
| ALL | B56-2 | 0.0004 | 0.5806 | 0.0000 |
| ALL | α | 0.0000 | 0.0000 | 0.0000 |
| ALL | β | 0.0000 | 0.0000 | 0.0000 |
| ALL | γ | 0.0000 | 0.0000 | 0.0000 |
| ALL | δ | 0.0000 | 0.3783 | 0.0000 |
| ALL | δ/γ | 0.0000 | 0.0001 | 0.0000 |
| ALL | ε | 0.0000 | 0.0000 | 0.0000 |
| B56-1 | B56-2 | 0.0073 | 0.0084 | 0.0004 |
| B56-1 | α | 0.0402 | 0.0001 | 0.1427 |
| B56-1 | β | 0.0000 | 0.0007 | 0.0000 |
| B56-1 | γ | 0.0000 | 0.0000 | 0.0078 |
| B56-1 | δ | 0.0007 | 0.3590 | 0.0001 |
| B56-1 | δ/γ | 0.0541 | 0.0073 | 0.9404 |
| B56-1 | ε | 0.0000 | 0.0008 | 0.0000 |
| B56-2 | α | 0.0245 | 0.0000 | 0.5771 |
| B56-2 | β | 0.0000 | 0.0001 | 0.0000 |
| B56-2 | γ | 0.0000 | 0.0000 | 0.0001 |
| B56-2 | δ | 0.0037 | 0.7509 | 0.0000 |
| B56-2 | δ/γ | 0.0225 | 0.0022 | 0.5209 |
| B56-2 | ε | 0.0000 | 0.0000 | 0.0000 |
| α | β | 0.0001 | 0.3957 | 0.0000 |
| α | γ | 0.0000 | 0.1756 | 0.0003 |
| α | δ | 0.2410 | 0.0056 | 0.0000 |
| α | δ/γ | 0.5949 | 0.5949 | 0.9106 |
| α | ε | 0.0000 | 0.6394 | 0.0000 |
| β | γ | 0.5956 | 0.0796 | 0.0019 |
| β | δ | 0.0030 | 0.0111 | 0.0044 |
| β | δ/γ | 0.0339 | 0.3860 | 0.0000 |
| β | ε | 0.0034 | 0.1216 | 0.0040 |
| γ | δ | 0.0025 | 0.0003 | 0.5038 |
| γ | δ/γ | 0.0563 | 0.8307 | 0.0060 |
| γ | ε | 0.0000 | 0.5501 | 0.0000 |
| δ | δ/γ | 0.9472 | 0.0098 | 0.0000 |
| δ | ε | 0.0000 | 0.0003 | 0.0000 |
| δ/γ | ε | 0.0000 | 0.3133 | 0.0000 |
