# Supplementary material for: A Nonsynonymous/Synonymous Substitution Analysis of the B56 Gene Family Aids in Understanding B56 Isoform Diversity
Source: PLoS One. 2015 Dec 21;10(12):e0145529. doi: 10.1371/journal.pone.0145529 (PMC4687035; doi:10.1371/journal.pone.0145529)
Supplement: S8 Table — The means and standard deviations from dN/dS analyses for the family-wide, B56-1, B56-2, and individual isoform groupings are provided. (DOCX) [file pone.0145529.s015.docx]

|  | dN | | dS | | dN/dS | |
| --- | --- | --- | --- | --- | --- | --- |
|  | mean | std | mean | std | mean | std |
| all  B56-1(αβε)  B56-2(γδ)  α  β  γ  δ  δ/γ  ε | 0.6909  0.5527  0.3351  0.2251  0.4347  0.1838  0.1597  0.1761  0.0339 | 0.3756  0.3146  0.1998  0.1590  0.2902  0.1046  0.2062  0.1260  0.0600 | 1.7267  1.3343  1.6497  0.9896  0.8663  1.1354  1.3590  1.2439  0.4096 | 1.0661  0.9980  1.0843  0.6738  0.4400  1.2348  0.9592  1.3106  0.2838 | 0.3817  0.4114  0.1697  0.1264  0.4746  0.1948  0.0505  0.1635  0.0116 | 0.2865  0.3976  0.1038  0.1212  0.3560  0.0732  0.0601  0.0680  0.0428 |
