# Supplementary material for: A Nonsynonymous/Synonymous Substitution Analysis of the B56 Gene Family Aids in Understanding B56 Isoform Diversity
Source: PLoS One. 2015 Dec 21;10(12):e0145529. doi: 10.1371/journal.pone.0145529 (PMC4687035; doi:10.1371/journal.pone.0145529)
Supplement: S9 Table — p values from dN/dS analyses for the family-wide, B56-1, B56-2, and individual isoform groupings are provided. p values less than 0.05 are highlighted in yellow. (DOCX) [file pone.0145529.s016.docx]

| **QUERY** | **SUBJECT** | **dN** | **dS** | **dN/dS** |
| --- | --- | --- | --- | --- |
| ALL | B56-1 | 0.0000 | 0.0000 | 0.0315 |
| ALL | B56-2 | 0.0000 | 0.7055 | 0.0000 |
| ALL | α | 0.0000 | 0.0000 | 0.0000 |
| ALL | β | 0.0001 | 0.0000 | 0.4725 |
| ALL | γ | 0.0000 | 0.0000 | 0.0000 |
| ALL | δ | 0.0000 | 0.0227 | 0.0000 |
| ALL | δ/γ | 0.0000 | 0.0156 | 0.0000 |
| ALL | ε | 0.0000 | 0.0000 | 0.0000 |
| B56-1 | B56-2 | 0.0000 | 0.0030 | 0.0000 |
| B56-1 | α | 0.0000 | 0.1253 | 0.0001 |
| B56-1 | β | 0.3753 | 0.0071 | 0.0905 |
| B56-1 | γ | 0.0000 | 0.0265 | 0.0035 |
| B56-1 | δ | 0.0000 | 0.8590 | 0.0000 |
| B56-1 | δ/γ | 0.0001 | 0.1826 | 0.0018 |
| B56-1 | ε | 0.0000 | 0.0000 | 0.0000 |
| B56-2 | α | 0.0009 | 0.0010 | 0.0419 |
| B56-2 | β | 0.0002 | 0.0000 | 0.0000 |
| B56-2 | γ | 0.0181 | 0.0024 | 0.3272 |
| B56-2 | δ | 0.0000 | 0.0817 | 0.0000 |
| B56-2 | δ/γ | 0.0176 | 0.0435 | 0.5099 |
| B56-2 | ε | 0.0000 | 0.0000 | 0.0000 |
| α | β | 0.0000 | 0.5957 | 0.0000 |
| α | γ | 0.0226 | 0.5526 | 0.0016 |
| α | δ | 0.0718 | 0.0689 | 0.0013 |
| α | δ/γ | 0.0353 | 0.8044 | 0.0245 |
| α | ε | 0.0000 | 0.0006 | 0.0000 |
| β | γ | 0.0018 | 0.5585 | 0.0001 |
| β | δ | 0.0000 | 0.0352 | 0.0000 |
| β | δ/γ | 0.0010 | 0.7165 | 0.0001 |
| β | ε | 0.0000 | 0.0000 | 0.0000 |
| γ | δ | 0.0001 | 0.1550 | 0.0000 |
| γ | δ/γ | 0.2283 | 0.7033 | 0.1257 |
| γ | ε | 0.0000 | 0.0011 | 0.0000 |
| δ | δ/γ | 0.0010 | 0.2676 | 0.0000 |
| δ | ε | 0.0000 | 0.0000 | 0.0000 |
| δ/γ | ε | 0.0000 | 0.0274 | 0.0000 |
